# Arachidin-1, a Prenylated Stilbenoid from Peanut, Enhances the Anticancer Effects of Paclitaxel in Triple-Negative Breast Cancer Cells

**DOI:** 10.3390/cancers15020399

**Published:** 2023-01-07

**Authors:** Sepideh Mohammadhosseinpour, Alexx Weaver, Meenakshi Sudhakaran, Linh-Chi Ho, Tra Le, Andrea I. Doseff, Fabricio Medina-Bolivar

**Affiliations:** 1 Molecular Biosciences Graduate Program, Arkansas State University, Jonesboro, AR 72401, USA; 2 Arkansas Biosciences Institute, Arkansas State University, Jonesboro, AR 72401, USA; 3 Molecular, Cellular, and Integrative Physiology Graduate Program, Michigan State University, East Lansing, MI 48824, USA; 4 Department of Physiology, and Department of Pharmacology and Toxicology, Michigan State University, East Lansing, MI 48824, USA; 5 Department of Biological Sciences, Arkansas State University, Jonesboro, AR 72401, USA

**Keywords:** triple-negative breast cancer, peanut, hairy roots, prenylated stilbenoids, resveratrol, arachidin-1, arachidin-3, paclitaxel, apoptosis, adjuvant

## Abstract

**Simple Summary:**

Triple-negative breast cancer (TNBC) is an aggressive type of cancer that is challenging to treat due to the lack of hormonal receptors used to target cancer cells; due to this, TNBC patients have high mortality rates. Plant-derived compounds are being sought as potential adjuvants for common chemotherapy drugs, such as paclitaxel (Pac). To this end, this research aimed to study the prenylated stilbenoid arachidin-1 (A-1) as a potential adjuvant for Pac. Here, the cytotoxic and apoptosis induction effects of A-1 alone and combined with Pac were investigated in 2D TNBC cell cultures and a 3D TNBC spheroid model. The results illustrated that A-1, in low micromolar concentrations, combined with Pac, inhibited cell proliferation, induced apoptosis, and reduced spheroid growth. Furthermore, our findings suggest that A-1 combined with Pac has the potential to be developed as a novel plant-derived treatment for TNBC.

**Abstract:**

Triple-negative breast cancer (TNBC) is one of the deadliest forms of breast cancer. Investigating alternative therapies to increase survival rates for this disease is essential. To this end, the cytotoxic effects of the prenylated stilbenoids arachidin-1 (A-1) and arachidin-3 (A-3), and non-prenylated resveratrol (RES) were evaluated in human TNBC cell lines as potential adjuvants for paclitaxel (Pac). A-1, alone or in combination with Pac, showed the highest cytotoxicity in TNBC cells. Apoptosis was further evaluated by measuring key apoptosis marker proteins, cell cycle arrest, and intracellular reactive oxygen species (ROS) generation. Furthermore, the cytotoxic effect of A-1 combined with Pac was also evaluated in a 3D spheroid TNBC model. The results showed that A-1 decreased the Pac IC_50_ approximately 2-fold in TNBC cells. The synergistic combination of A-1 and Pac arrested cells in G2/M phase and activated p53 expression. In addition, the combined treatment increased intracellular ROS generation and induced apoptosis. Importantly, the combination of A-1 with Pac inhibited TNBC spheroid growth. Our results demonstrated that A-1 in combination with Pac inhibited cell proliferation, induced apoptosis through mitochondrial oxidative stress, and reduced TNBC spheroid growth. These findings underscore the impactful effects of the prenylated stilbenoid A-1 as a novel adjuvant for Pac chemotherapy in TNBC treatment.

## 1. Introduction

The 2020 world cancer statistics showed approximately 2.26 million new cases of breast cancer, making breast cancer the most commonly diagnosed cancer in females [1]. Within the United States, one in eight women will develop invasive breast cancer within their lifetime [2,3]. Breast cancers are divided into four major phenotypic groups based on the presence or absence of key biological markers, namely estrogen receptor (ER), progesterone receptor (PR), and human epidermal growth factor receptor 2 (HER2) [4]. Triple-negative breast cancer (TNBC) is an aggressive breast cancer lacking all three hallmark hormonal receptors. TNBC is refractory to endocrine or HER2 treatment, and regimens of targeted therapies remain undeveloped. In comparison to other breast cancer subtypes, patients with TNBC have a shorter survival time, higher mortality rate, and worse clinical outcomes [4]. The main treatment for TNBC is chemotherapy, but the effectiveness of standard postoperative adjuvant chemoradiotherapy is poor and associated with adverse effects. Considering this, the introduction of combination treatment options, compensating for lower drug dosage, should be considered to reduce these adverse effects. 

Paclitaxel (Pac) is a widely used anticancer drug that is isolated from the bark and trunk of the Pacific yew tree or semi-synthesized from 10-deacetylbaccatin III, a renewable compound extracted from the needles of yew species [5]. Pac acts by inhibiting tubulin depolymerization in spindles leading to cell cycle arrest, reactive oxygen species (ROS) accumulation, and apoptosis [6]. However, the side effects associated with using Pac as a cancer treatment are highly undesirable. Known side effects include: peripheral neuropathy; fatigue, anxiety, and depression, decreased white blood cell count; diarrhea, vomiting, anemia, edema [7,8]. These side effects often lead to more problems in cancer patients, such as increased infections due to a decrease in white blood cell counts. 

Improved cancer chemosensitivity while minimizing undesirable side effects is needed to improve quality of life and therapeutic outcomes for TNBC patients. Plant natural products, including stilbenoids, have attracted great attention as potential anticancer treatments or adjuvants [9,10]. Stilbenoids are polyphenols produced in certain plants, such as grapes and peanuts, and function as phytoalexins to protect against fungal infection. Resveratrol (RES) is the most studied non-prenylated stilbenoid. It has shown anticancer activity in many different cancers including of the breast [5,11]. 

**Figure 1:**
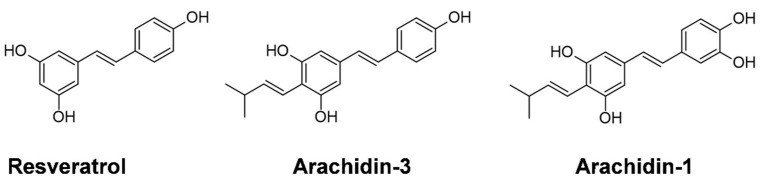
Chemical structure of non−prenylated stilbenoid (resveratrol) and prenylated stilbenoids (arachidin−3 and arachidin−1).

Treatment of breast cancer with RES has shown some limitations including low bioavailability [12]. Prenylated stilbenoids such as arachidin-1 (A-1) and arachidin-3 (A-3) (Figure 1) have shown potential higher bioavailability due to their slower metabolization. In vitro studies using recombinant UGTs and human liver and intestine microsomes demonstrated significantly lower rates of glucuronidated products from A-1 and A-3 in comparison to their non-prenylated analogues piceatannol and RES, respectively [13]. Furthermore, prenyl groups occurring in A-1 and A-3 increase their lipophilicity compared to RES and thus may boost cell membrane interactions for better absorption and increased metabolism. Even though there are a few studies available regarding A-1 and A-3 metabolism, no specific research has been carried out on their absorption, distribution, or elimination. 

A-1 demonstrated antioxidative, anti-inflammatory, and anti-tumorigenesis properties in human leukemia HL-60 cells [14,15,16,17]. A-1, when compared to RES and A-3, was found to have significantly higher cytotoxicity in HL-60 cells. Studies with A-1 in combination with the antidiabetic drug metformin showed that A-1 sensitized non-small cell lung cancer cells [18]. Recently, our research group described the anti-cancer properties of A-1 and A-3 in TNBC cells MDA-MB-231 and MDA-MB-436 [19]. A-1 showed the highest cytotoxic effects and promoted apoptosis through the intrinsic pathway. Importantly, A-1 was not cytotoxic to the non-cancerous breast epithelial cell line MCF-10A [19]. 

Studies with A-1 have been challenging because of its limited availability. To address this issue, hairy root cultures of peanut were developed as an elicitor-controlled bioproduction platform for A-1 and other prenylated stilbenoids. Upon elicitation, high quantities of prenylated stilbenes (>700 mg/L) can be extracted from the culture medium and further purified for testing in bioassays [20,21]. 

The aim of this study was to assess the cytotoxic, oxidative stress, cell cycle arrest, and apoptosis induction capabilities of peanut hairy root culture-derived A-1 and A-3 and commercially procured RES alone and in combination with Pac. The most cytotoxic compound was also evaluated in combination with Pac in a TNBC spheroid model. 

## 2. Materials and Methods

### 2.1. Cell Lines and Reagents

The TNBC cells MDA-MB-231 (ATCC HTB-26™; Manassas, VA, USA) and MDA-MB-436 (ATCC HTB-130™; Manassas, VA, USA), as well as the non-cancerous epithelial cell line MCF-10A (ATCC CRL-10317™; Manassas, VA, USA), were obtained from the American Type and Culture Collection (ATCC; Manassas, VA, USA). MCF-10A cells were maintained in Dulbecco’s Modified Eagle’s Medium (DMEM)/F12 medium supplemented with a MEGM Kit (Lonza Pharma and Biotech; Basel, Switzerland) at 37 °C, with a 5% CO_2_ humidified environment. MDA-MB-231 cells were cultured in Dulbecco’s Modified Eagle’s Medium (DMEM) (ATCC; Manassas, VA, USA) at 37 °C, with a 5% CO_2_ humidified environment. MDA-MB-436 cells were cultured in Leibovitz’s L-15 medium (ATCC; Manassas, VA, USA) with 10 µg/mL of insulin (Gibco; Life Technologies; Grand Island, NY, USA) and 16 µg/mL of glutathione (Sigma-Aldrich; St. Louis, MO, USA) at 37 °C in high humidity, with 0% CO_2_. TNBC cell line growth media (DMEM and Leibovitz’s L-15 media) included 10% fetal bovine serum and 1% penicillin–streptomycin solution (100 IU/mL of penicillin and 100 µg/mL of streptomycin; ATCC; Manassas, VA, USA). The prenylated stilbenoids A-1 and A-3 were purified from elicited peanut hairy root cultures as previously described [19,21]. RES and Pac were purchased from Sigma-Aldrich (St. Louis, MO, USA). RES, Pac, A-1, and A-3 were dissolved in dimethyl sulfoxide (DMSO) (ATCC; Manassas, VA, USA).

### 2.2. Cytotoxicity Assays

Proliferation assays were performed with RealTime-Glo^TM^ MT Cell Viability Assay (Promega, Madison, WI, USA). Cells (2 × 10^4^ cells/well) were seeded, in triplicate, with 100 µL of media in 96-well plates; following seeding, plates were incubated for 24 h at 37 °C with 5% or 0% CO_2_ depending on the cells cultured. Pac concentrations ranged from 0.1 nM to 1000 nM; RES, A-1, and A-3 concentrations ranged from 0.1 µM to 1000 µM. Cells treated with 0.1% DMSO (ATCC, Manassas, VA, USA) were used as controls. Luminescence was measured at 24 h, 48 h, and 72 h using a Cytation^TM^ 5 (BioTek, Winooski, VT, USA) plate reader. Data were analyzed using GraphPad Prism 9 (San Diego, CA, USA). 

### 2.3. Caspase-3/7 Activity Assays

The Apo-ONE^®^ Homogenous Caspase-3/7 Assay (Promega, Madison, WI, USA) was used to determine caspase-3/7 activity following the manufacturer’s protocol. Cells (10^4^ cells/well) were seeded into 96-well plates and incubated at 37 °C for 24 h followed by 48 h treatments. Cells treated with 0.01% DMSO were the control group. Throughout the treatment period, all plates were incubated at 37 °C with 5% or 0% CO_2_ depending on the cell line. Subsequently, cells were incubated in the presence of the caspase reagent at room temperature for 1 h in the dark, and fluorescence was measured using the Cytation^TM^ 5 (BioTek; Winooski, VT, USA) plate reader [excitation 485 ± 20 nm and an emission 530 ± 25 nm].

### 2.4. Reactive Oxygen Species (ROS)

The production of ROS was evaluated using 2′,7′-dichlorodihydrofluorescein diacetate (H_2_DCFDA) (Thermo Fisher Scientific; Waltham, MA, USA). Cells (2 × 10^5^ cells/well) were seeded with 100 µL media in 96-well plates. After incubation at 37 °C for 24 h, the cells were treated with A-1 or Pac alone or A-1 in combination with Pac and incubated for an additional 24 h. Cells incubated with 0.01% DMSO were used as controls. After treatment, cells were washed with warm 1X phosphate-buffered saline (PBS) and stained with 20 µM H_2_DCFDA at 37 °C for 30 min. Fluorescence measurements were taken using a Cytation^TM^ 5 (BioTek, Winooski, VT, USA) plate reader [excitation 492–495 nm and emission 517–527 nm] every 6 h for 48 h. The average value was calculated from the RFU (relative fluorescence units) of the treated group normalized by a control group (DMSO). 

For flow cytometry analysis, cells (2 × 10^5^ cells/well) were seeded into 6-well plates. After 24 h incubation, cells were treated and incubated for an additional 24 h. Then, the cells were stained with 20 µM H_2_DCFDA at 37 °C for 30 min. Following staining, the fluorescence intensity of DCF in each group was analyzed by flow cytometry using BD FACS Aria (BD Biosciences, Franklin Lakes, NJ, USA) and the data were analyzed using BD FACSDiva software. The data were compared to cells treated with 0.01% DMSO. Cells treated with H_2_O_2_ (10 µM and 100 µM) were used as the positive control. 

### 2.5. Cell Cycle Analyses 

Cell cycle progression was evaluated using propidium iodide (PI) staining. Cells (10^6^ cells/well) were seeded into 6-well plates, in triplicate. After 24 h incubation, cells were treated with various concentrations of A-1 (0.2 µM and 2 µM), Pac (0.2 nM and 2 nM) as well as the four combinations A-1 (2 µM)/Pac (2 nM), A-1 (2 µM)/Pac (0.2 nM), A-1 (0.2 µM)/Pac (2 nM), A-1 (0.2 µM)/Pac (0.2 nM); and incubated for an additional 24 h. Cells cultured in medium containing 0.01% DMSO were used as controls. MDA-MB-231 cells were harvested by trypsinization; MDA-MB-436 cells were harvested by mechanically detaching with a cell scraper. Collected cells were immediately fixed in ice-cold 70% ethanol, added in a dropwise manner while vortexing, and incubated at 4 °C for 1 h. Afterward, the cells were washed with cold 1X PBS, treated with 1 mg/mL of ribonuclease A, and incubated for 30 min at 37 °C. After incubation, 10 µg/mL of PI was added and cells were incubated for an additional 30 min at room temperature; cells were observed via flow cytometry using a BD FACS Aria Sorter (BD Biosciences, Franklin Lakes, NJ, USA). Data were analyzed by Modfit LT 5.0 software (Verity Software House; Topsham, ME, USA). 

### 2.6. Detection of Apoptosis Using Annexin V-FITC Assays

Cellular apoptosis progression was observed using Annexin V-FITC staining. Cells (10^6^ cells/well) were seeded into 6-well plates and incubated at 37 °C for 24 h. This was followed by treatment with A-1 or Pac alone or A-1 combined with Pac for an additional 24 h. Cells incubated with 0.01% DMSO (ATCC; Manassas, VA, USA) were used as controls. The cells were stained with Annexin V-FITC and PI at room temperature for 15 min following the manufacturer’s instructions (BD Life Sciences; Ashland, OR, USA); stained cells were analyzed by flow cytometry using BD FACS Aria (BD Biosciences, Franklin Lakes, NJ, USA). Populations in the Annexin V positive/PI negative staining quadrant signified early apoptotic cells, while populations in the Annexin V positive/PI positive quadrant indicated late apoptosis/necrotic cells. Data were analyzed with FlowJo™ v10.8 Software (BD Life Sciences; Ashland, OR, USA).

### 2.7. Immunoblotting

Cells (5 × 10^6^ cells/3 mL medium) were cultured in 6-well plates for 24 h, followed by a 24 h treatment period. Protein cell lysates were obtained using RIPA Lysis and Extraction Buffer plus Halt™ Protease Inhibitor Cocktail (Thermo Fisher Scientific; Waltham, MA, USA) and stored at −20 °C. For western immunoblotting, membranes were blocked with blocking buffer [1X TBS combined with 1% Tween 20 (1X TBST) (Bio-Rad; Hercules, CA, USA)] containing 5% powdered non-fat dry milk for 1 h at room temperature. After blocking, membranes were washed with 1X TBST and incubated overnight at 4 °C in primary antibody solution. Original blots can be found at Supplementary File S1. Primary antibodies were rabbit anti-caspase-8 (D35G2) mAb (1:500; Cell Signaling Technology; Danvers, MA, USA), rabbit anti-PARP mAb (1:500; Cell Signaling Technology; Danvers, MA, USA), mouse anti-human survivin (6E4) mAb (1:500; Cell Signaling Technology; Danvers, MA, USA), mouse anti-human caspase-9 mAb (1 µg/mL; R&D Systems; Minneapolis, MN, USA), mouse anti-vinculin antibody (2 µg/mL; R&D Systems; Minneapolis, MN, USA), and mouse anti-human GAPDH antibody (0.05 µg/mL; R&D Systems; Minneapolis, MN, USA), rabbit anti-caspase-7 mAb (1:500; Cell Signaling Technology; Danvers, MA, USA) and mouse anti-human p53 mAb (1 µg/mL; R&D Systems; Minneapolis, MN, USA). 

Following primary antibody incubation, membranes were washed with 1X TBST and a secondary antibody solution was added. The secondary antibodies used were: HRP-conjugated goat anti-rabbit IgG (1:2000; Cell Signaling Technology; Danvers, MA, USA) and HRP-conjugated AffiniPure goat anti-mouse IgG secondary antibody (1:50,000; Jackson Immunoresearch Laboratories; West Grove, PA, USA). Membranes were incubated with secondary antibodies at room temperature for 1 h. Membranes were subsequently washed with 1X TBST before treatment with SuperSignal™ West Pico PLUS Chemiluminescent Substrate (Thermo Fisher Scientific; Waltham, MA, USA). Protein expression was observed using the Li-COR Odyssey^®^ Fc Imaging System (LI-COR Biosciences; Lincoln, NE, USA) and data were analyzed using ImageJ software (NIH, Bethesda, MD).

### 2.8. 3-Dimensional Culture

Human TNBC MDA-MB-231 cells were grown into spheroids as previously described [22]. Briefly, cells (passage number < 20) were seeded at a density of 2000 cells/well into V-bottom 96-well plates (Greiner bio-one, #651101, Frickenhausen, Germany), centrifuged at 500 g for 5 min, and supplemented with complete media containing 2.5% Matrigel (Corning, Glendale, AZ). Spheroids were cultured for up to 6 days prior to treatment. Cell viability was determined using CellTiter-Glo^®^ 3D Cell Viability Assay (Promega) following the manufacturer’s instructions. The luminescence was measured using Synergy™ Neo2 Multi-Mode Microplate Reader (Agilent BioTek, CA). The percentage of viable cells was calculated as (Y_treatment_ − Y_blank_)/(Y_control_ − Y_blank_) × 100%, where Y is the luminescence. In addition, cell viability and death were assessed by staining the spheroids with 2 μM calcein AM (Invitrogen, Carlsbad, CA) and 2 μg/mL PI (Molecular Probes, Eugene, OR) for 1 h at 37 °C, as previously described [22]. The diameter of the spheroids was assessed by Z-stack images (30 μm thick, accounting for 10–15 stacks per spheroid) acquired using a Cytation^TM^ 3 microscope (Agilent BioTek, CA). The diameter and PI mean fluorescence intensity were quantified using ImageJ software (NIH, Bethesda, MD). Changes in spheroid growth were calculated as the difference in the diameters between the final and initial days of the treatment (Δ diameter). 

### 2.9. Statistical Analyses

Data were expressed as the mean ± standard error of the mean (SEM). When measuring the mean difference between two groups, students’ t-test analyses were used (Tables 1 and 2, Figures 3, 7, 10 and 11) and one-way analysis of variance (ANOVA) was used when comparing the significance of more than two groups (Figures 4, 5, 6, 8 and 9). All statistical analyses were performed using GraphPad Prism 9 (San Diego, CA, USA). Calculations with a *p*-value of *p* < 0.05 were deemed statistically significant.

## 3. Results

### 3.1. Stilbenoids Increased the Cytotoxicity of Pac in TNBC Cells

To determine if stilbenoids increased the cytotoxicity of Pac in human TNBC cells, checkerboard drug combination assays were performed. For this purpose, cells were treated with Pac at concentrations from 0.1 nM to 1000 nM and RES, A-1, and A-3 at concentrations from 0.1 µM to 1000 µM for 24, 48, and 72 h. In both cell lines, the combination of A-3 or RES with Pac did not show a significant change in the IC_50_ values when the Pac concentration increased (Table 1 and Table S1). Importantly, A-1 showed a synergistic interaction with Pac in which increasing the concentration of A-1 or Pac resulted in an approximately 2-fold decrease in the IC_50_ of Pac or A-1, respectively (Table 2). In addition to these observations, the IC_50_ values of A-1 were at least 3-fold lower than RES and A-3 (Table 1 and Table S1); there were no significant changes in the IC_50_ of A-1 combined with Pac between the three treatment time points. This demonstrates that A-1 augments the cytotoxic effect of Pac in TNBC cells; therefore, this study focused on this combination for the following analysis.

### 3.2. A-1 Combined with Pac Activates Caspase-3/7

**Figure 2:**
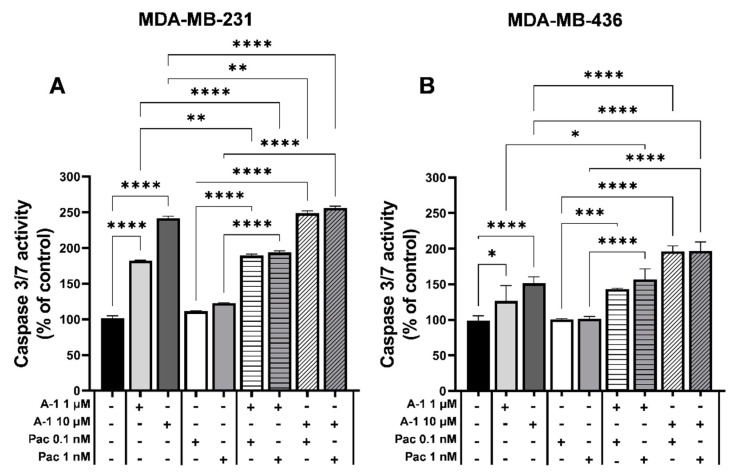
Arachidin−1 (A−1) increases paclitaxel (Pac) induced apoptosis. MDA−MB−231 (**A**) and MDA−MB−436 (**B**) cells were treated with A−1 (1 and 10 µM), Pac (0.1 and 1 nM), or a combination treatment for 48 h. Caspase 3/7 activity was estimated utilizing the Apo−ONE^®^ Homogenous Caspase-3/7 Assay. Cells treated with 0.01% DMSO were used as a control. Data represent mean ± SEM. N = 3. * *p* < 0.05, ** *p* < 0.01, *** *p* < 0.001, **** *p* < 0.0001 versus control.

Since A-1 combined with Pac at low concentrations suppressed growth in TNBC cells, the potential mechanism underlying cell growth inhibition was determined by measuring caspase-3 and caspase-7 activity; these proteins are effector caspases for both intrinsic and extrinsic apoptosis pathways. MDA-MB-231 and MDA-MB-436 cells were treated with A-1 (1 and 10 µM) and Pac (0.1 and 1 nM) for 48 h (the optimal time point for this experiment). For MDA-MB-231, when comparing cells treated with A-1 1 µM or 10 µM alone to cells treated with A-1 combined with Pac, the combination treatments demonstrated significant 7% and 13% increases in caspase 3/7 activity, respectively (Figure 2A). Similarly, when comparing cells treated with A-1 1 µM or 10 µM alone to cells treated with A-1 combined with Pac, the combination treatment statistically significantly increased caspase 3/7 activity in a dose-dependent manner from roughly 16% to 45%, respectively, in MDA-MB-436 (Figure 2B). Additionally, the combination treatment compared to Pac alone led to a statistically significant augmentation of caspase 3/7 activity in a dose-dependent manner from approximately 78% to 134% and 42% to 96% in MDA-MB-231 and MDA-MB-436, respectively (Figure 2A,B). Therefore, these results highlighted that A-1 combined with Pac synergistically increased the caspase 3/7 activity and posed as a potential mechanism underlying the cytotoxic effect of A-1 combined with Pac. 

### 3.3. Effect of A-1 Combined with Pac in a Non-Cancerous Cell Line

**Figure 3:**
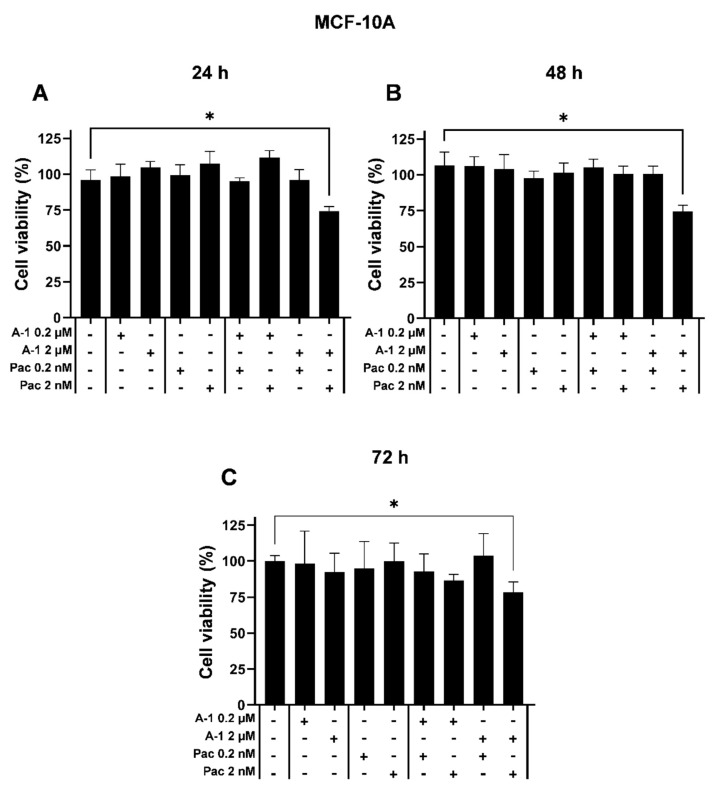
Cytotoxicity of arachidin−1 (A−1) combined with paclitaxel (Pac) in MCF−10A cells. Cell viability was evaluated with Pac or A−1 alone or in combination for (**A**) 24, (**B**) 48, and (**C**) 72 h. DMSO was used as diluent control. Data represent mean ± SEM. N = 3. * *p* < 0.05 versus control.

To study if A-1 combined with Pac was cytotoxic to human non-cancerous breast epithelial MCF-10A cells, cells were treated with A-1 (0.2 and 2 µM) and Pac (0.2 and 2 nM) as well as their respective combinations. The higher concentration values for each compound were chosen based on their IC_50_ concentration, which was approximately 2 µM in TNBC cells, whereas the lower concentration was 10-fold lower than the IC_50_ value and it approximately represented the lowest effective concentration seen in the TNBC cells. Cytotoxic effects were observed only at the highest concentrations of the combined treatment (A-1 (2 µM) and Pac (2 nM)), reducing viability by ~25% at 24, 48, and 72 h (Figure 3). Importantly, A-1 at low concentration (1 µM) combined with Pac at low concentration (1 nM) decreased the percentage of cell viability by more 50% in both TNBC cell lines (Figure S1). These results demonstrated that A-1 at approximately IC_50_ value concentration (2 µM) and 10-fold lower combined with Pac at approximately IC_50_ value concentration (2 nM) and 10-fold lower did not show cytotoxicity to non-cancerous cells (Figure 3). These concentrations were selected for subsequent assays in TNBCs.

### 3.4. Pac Combined with A-1 Induces ROS in TNBC Cells

**Figure 4:**
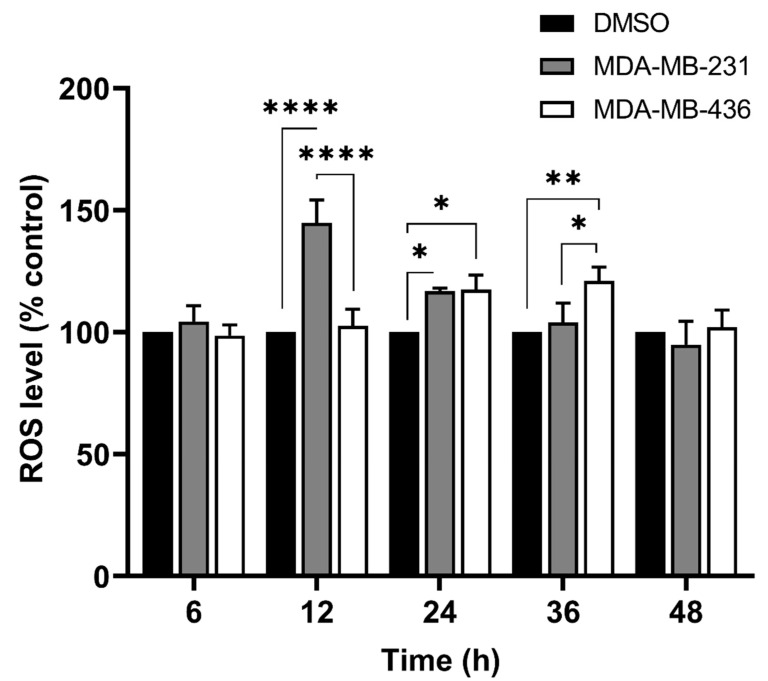
Intracellular ROS generated in MDA−MB−231 (gray bar) and MDA−MB−436 (white bar). Cells were treated with arachidin−1 (0.2 µM) at varying time points (6, 12, 24, 36, and 48 h); cells treated with 0.01% DMSO were used as controls (black bar). After treatment with arachidin−1, cells were stained with H_2_DCFDA and fluorescence was measured (excitation: 492–495 nm; emission: 517–527 nm). Data represent mean ± SEM. N = 3. * *p* < 0.05, ** *p* < 0.01, **** *p* < 0.0001 versus control.

Our previous study demonstrated that MDA-MB-231 and MDA-MB-436 TNBC cells activate apoptosis markers at different time points [19]. To investigate the effects of A-1 on these two TNBC cells, the intracellular ROS accumulation was measured in cells treated with A-1 (0.2 µM) at 6, 12, 24, 36, and 48 h incubation. The results revealed that the concentration of intracellular ROS increased steadily over the time points when compared to the control group (DMSO) from ~99% at 6 h to ~120% at 36 h and then decrease to ~101% at 48 h in MDA-MB-436 cells (Figure 4). In contrast, the percentage of ROS levels in MDA-MB-231 cells compared to the percentage of the controls increased immediately after 12 h treatment from ~104% at 6 h to ~145% and then decreased gradually to ~95% at 48 h (Figure 4). MDA-MB-231 and MDA-MB-436 activated intracellular ROS production at different time points and both cell lines showed an approximately similar percentage of ROS activity from 24 h. We measured A-1 without H_2_DCFDA and compared it to the control with staining because A-1 has previously shown to exhibit fluorescence at excitation 335 nm and emission 374 nm, or absorbance at 320 nm [23]; we didn’t find any conflict between A-1 and H_2_DCFDA staining (Figure S2). Therefore, 24 h time point was selected for further flow cytometry assays. 

**Figure 5:**
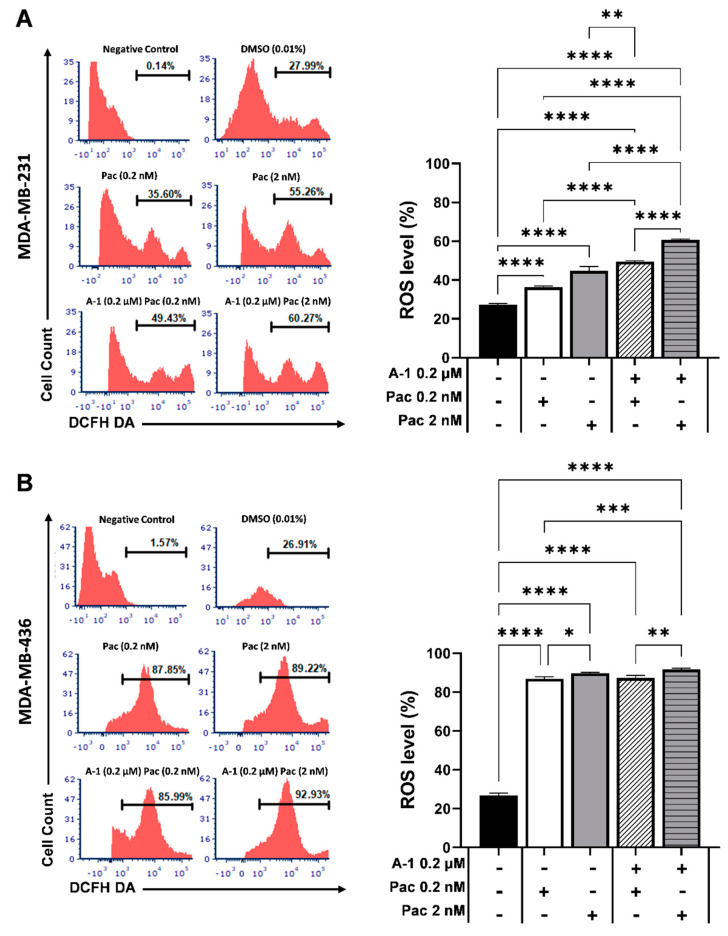
Effects of arachidin−1 (A−1) combined with paclitaxel (Pac) on intracellular ROS activity in TNBC cells. MDA−MB−231 (**A**) and MDA-MB-436 (**B**) cell lines were treated with A−1 (0.2 and 2 µM) and/or Pac (0.2 and 2 nM) for 24 h. Cells were stained with H_2_DCFDA and reactive oxygen species accumulation was determined via flow cytometry. The histogram data are represented as a bar graph mean ± SEM. N = 3. * *p* < 0.05, ** *p* < 0.01, *** *p* < 0.001, **** *p* < 0.0001 versus controls.

To investigate the effects of A-1 combined with Pac on intracellular ROS accumulation, MDA-MB-231 and MDA-MB-436 cells were treated with either Pac (0.2 and 2 nM) or the combination treatment with A-1 (0.2 µM) for 24 h. As shown in Figure 5, the combination treatment of A-1 and Pac enhanced the generation of intracellular ROS by ~13% (Figure 5A) and ~2% (Figure 5B) in both cell lines (MDA-MB-231 and MDA-MB-436, respectively) in comparison with treatment using Pac alone. Due to the small changes in the intracellular ROS level in MDA-MB-436, when compared to the moderate changes in MDA-MB-231, the 24 and 48 h time points were chosen for protein expression by western blot. 

### 3.5. A-1 Combined with Pac Induces G2/M Phase Cell Cycle Arrest in TNBC Cells

**Figure 6:**
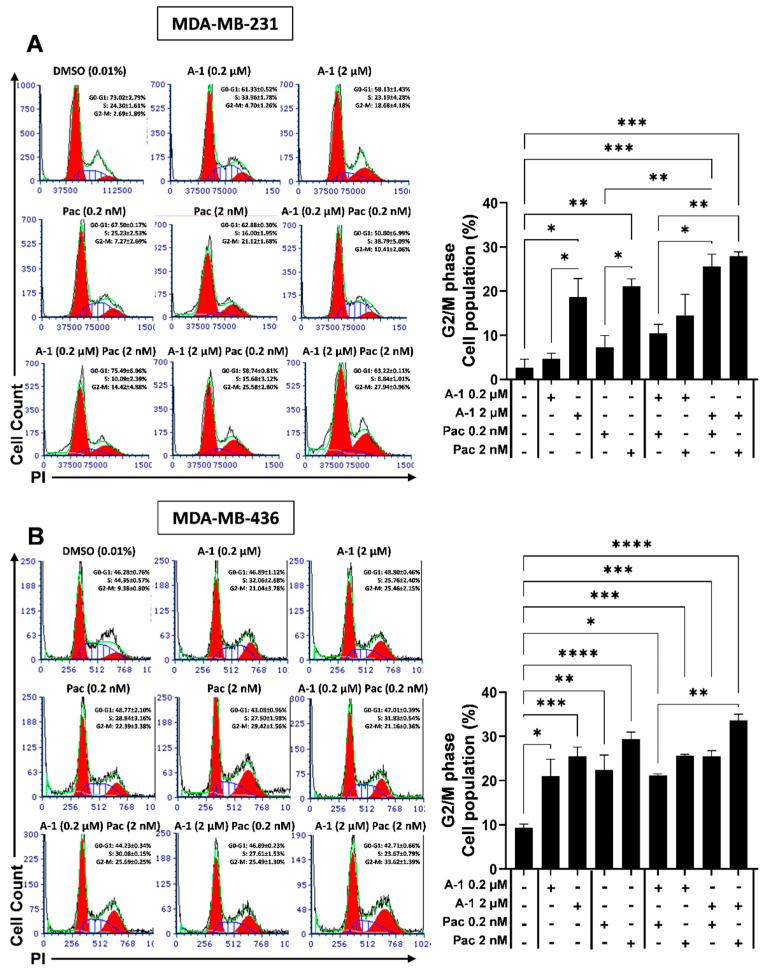
Arachidin−1 induced cell cycle arrest in MDA−MB−231 and MDA−MB−436 in G2/M phase. Representative histogram analysis and bar graph analysis of the cell cycle in MDA−MB−231 (**A**) and MDA−MB−436 (**B**). Cells were treated with A−1 (0.2 and 2 µM), Pac (0.2 and 2 nM), or a combination of these treatments for 24 h. After treatment, an estimation of the proportion of cells in each phase of the cell cycle was made using PI staining fluorescence collected via flow cytometry. Data were analyzed by FCS express 7 software (De Novo Software, USA). Data represent mean ± SEM. N = 3. * *p* < 0.05, ** *p* < 0.01, *** *p* < 0.001, **** *p* < 0.0001 versus control.

**Figure 7:**
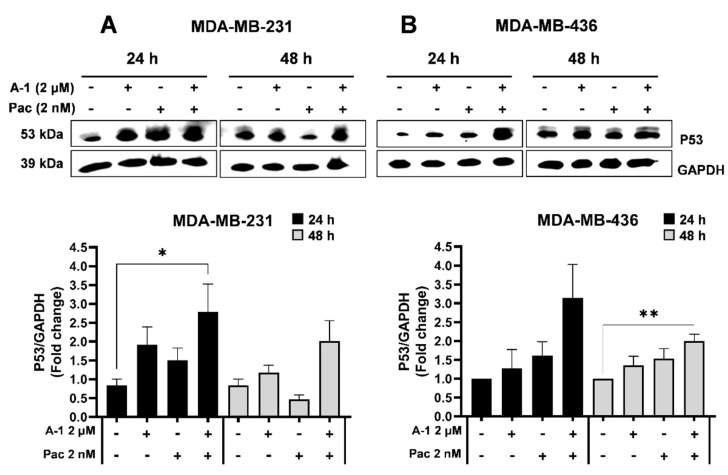
Arachin−1 treatment shows a trend of p53 upregulation in MDA−MB−231 and MDA−MB−426. Cells were treated with arachidin−1 (A−1; 2 μM), paclitaxel (Pac; 2 nM), or the two in combination. Cells treated with 0.01% DMSO were used as a control group for both the 24 h and 48 h time points. Cells were harvested at both time points and protein expression was determined via western blotting. Western blot images of p53 in MDA-MB-231 (**A**) and MDA-MB-436 (**B**) are shown with loading control GAPDH. Densiometric comparisons for MDA-MB-231 (**A**) and MDA-MB-436 (**B**) demonstrate the fold change of the target protein compared to the loading control GAPDH. Data represent mean ± SEM. N = 3. * *p* < 0.05, ** *p* < 0.01 versus control.

To further understand the mechanistic nature behind the cytotoxic effects of A-1 and Pac combination, cells treated with this combination were evaluated for cell cycle arrest. As shown in Figure 6, the treatment with either A-1 or Pac alone caused a statistically significant, dose-dependent increase by ~13% and ~4% of cells in the G2/M phase for MDA-MB-231 and MDA-MB-436 cells, respectively. However, the combination treatment of A-1 and Pac compared to A-1 (2 µM) alone enhanced the cell cycle arrest in G2/M phases by ~9% in MDA-MB-231 and ~4% in MDA-MB-436 cells (Figure 6). Additionally, combination treatment compared to Pac treatment alone increased the number of cells in G2/M phase by ~4–18% and ~3–4% in MDA-MB-231 and MDA-MB-436 cells, respectively. To determine if cell cycle arrest after treatment with Pac alone or in combination with A-1 was related to the expression of p53, cells were treated with A-1 (2 µM), Pac (2 nM), or a combination of the two for 24 h and 48 h. The data demonstrated that Pac alone or in combination with A-1 significantly increased the expression levels of p53 in comparison to the control group at 24 h in MDA-MB-231 and at 48 h in MDA-MB-436 (Figure 7).

### 3.6. Apoptosis Effect of A-1 and Pac alone or in Combination on TNBC Cells

**Figure 8:**
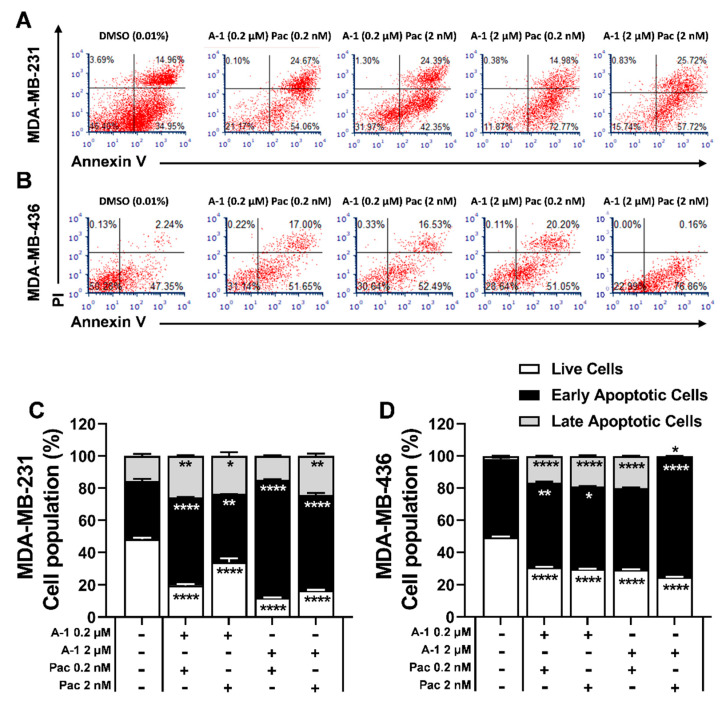
Arachidin−1 (A−1) induced apoptosis in MDA−MB−231 (**A**,**C**) and MDA−MB−436 (**B**,**D**). Cells were treated with A−1 (0.2 and 2 µM), paclitaxel (Pac; 0.2 and 2 nM), or a combination of these treatments for 24 h. Cells were stained with Annexin V−FITC/PI staining and apoptosis activity was measured. Data represent the mean ± SEM. N = 3. * *p* < 0.05, ** *p* < 0.01, **** *p* < 0.0001 versus control.

To investigate whether the growth inhibition and cell cycle arrest effects of A-1 combined with Pac were related to apoptosis, TNBC cells were treated with these compounds for 24 h and stained with PI and Annexin V. The combination treatment enhanced apoptosis in a dose-dependent manner in both TNBC cells. As shown in Figure 8, the percentage of cells in early apoptosis within the low-concentration combination (A-1 (0.2 µM)/Pac (0.2 nM)) compared to the high-concentration combination (A-1 (2 µM)/Pac (2 nM)) increased significantly by ~7% in MDA-MB-231 (Figure 8B) and by ~20% in MDA-MB-436 cells (Figure 8C). Thus, the results showed that combination treatments induce early apoptosis in a dose-dependent manner; additionally, the high-concentration combination showed increased apoptosis in comparison to the low-concentration combination. Therefore, caspase activity was further investigated with A-1 (2 µM) and Pac (2 nM).

### 3.7. A-1 Combined with Pac Induced Apoptosis through the Intrinsic Pathway in TNBC Cells

**Figure 9:**
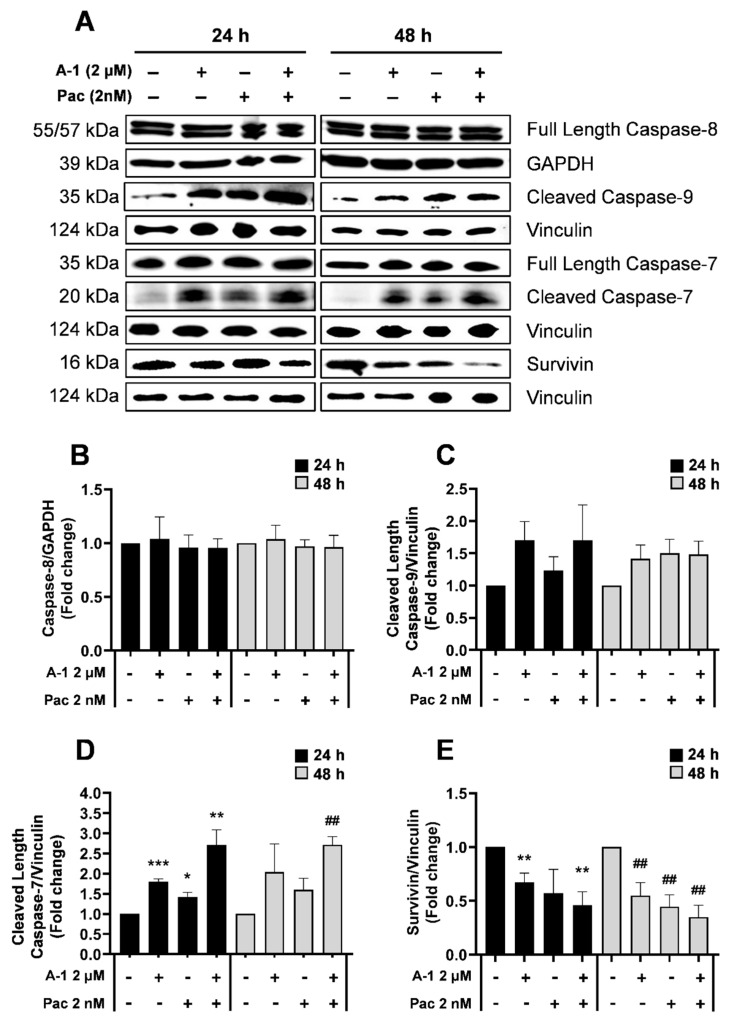
Apoptosis protein expression in treated TNBC cells. Western blot analysis of MDA−MB−231 (**A**) and the respective densiometric comparisons for caspase−8 (**B**), cleaved caspase−9 (**C**), cleaved caspase−7 (**D**), and survivin (**E**). Cells were treated with A−1 (0.2 and 2 µM) and/or Pac (0.2 and 2 nM), as well as DMSO as a control at 24 h & 48 h timepoints. Densiometric comparisons represent the fold change of the target protein compared to the loading control GAPDH or Vinculin. The data represent mean ± SEM. N = 3. * *p* < 0.05, ** *p* < 0.01, *** *p* < 0.001 versus control (24 h) and ## *p* < 0.01 versus control (48 h).

**Figure 10:**
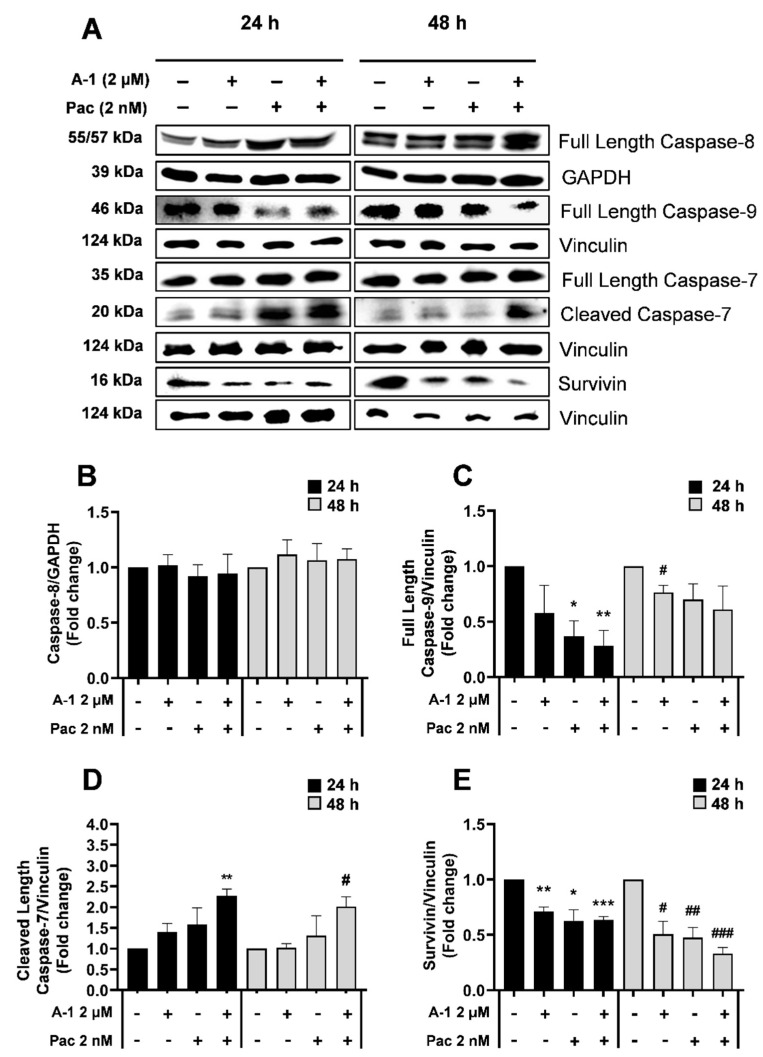
Apoptosis protein expression in treated TNBC cells. Western blot analysis of MDA−MB−436 (**A**) and the respective densiometric comparisons for caspase−8 (**B**), full-length caspase−9 (**C**), cleaved caspase−7 (**D**), and survivin (**E**). Cells were treated with A−1 (0.2 and 2 µM) and/or Pac (0.2 and 2 nM), as well as DMSO as a control at 24 h & 48 h time points. Densiometric comparisons represent the fold change of the target protein compared to the loading control GAPDH or Vinculin. The data represent the mean ± SEM. N = 3. * *p* < 0.05, ** *p* < 0.01, *** *p* < 0.001 versus control (24 h) and # *p* < 0.05, ## *p* < 0.01, ### *p* < 0.001 versus control (48 h).

Cells undergo apoptosis through two main pathways: intrinsic (mitochondrially mediated) and extrinsic (death receptor mediated). To determine which pathway was initiated following treatment with Pac alone or in combination with A-1, the expression levels of intrinsic and extrinsic apoptosis-related proteins were evaluated using western blotting. TNBC cells were treated with A-1 (2 µM), Pac (2 nM), or their combination for 24 h and 48 h. As revealed in Figure 9, there were no significant changes in full-length caspase 8 and full-length caspase-7 expression in MDA-MB-231 for either time point (Figure 9A,B and Figure S3A); however, there was a ~1.5-fold increase in the cleaved caspase-9 expression when comparing the combination treatment to each single treatment at 24 h (Figure 9C). Additionally, there were statistically significant upregulations in cleaved caspase-7 (~1.9 and 1.7-fold-increase) for the combination treatment in comparison to Pac alone at 24 h and 48 h, respectively (Figure 9D). MDA-MB-436 protein expression analysis indicated no significant fold changes in full-length caspase-8 and full-length caspase-7 expression for either time point (Figure 10B and Figure S3B). In MDA-MB-436, the combination treatment significantly reduced expression levels of full-length caspase-9 (~0.5-fold-decrease) and significantly increased the expression levels of cleaved caspase-7 (~2-fold-increase) at 24 h and 48 h, when compared to the single-treatment and control groups (Figure 10C,D). Additionally, there were gradual statistically significant fold change decreases (up to ~0.5-fold) in survivin protein levels in the combination treatments compared to the single-treatment and control groups in both MDA-MB-231 and MDA-MB-436 cell lines at 24 h and 48 h (Figure 9E and Figure 10E). These results indicate that A-1 combined with Pac potentially induces apoptosis in TNBC cells through the intrinsic pathway. 

### 3.8. A-1 Enhances the Efficacy of Pac in TNBC Spheroids by Inducing Cell Death

**Figure 11:**
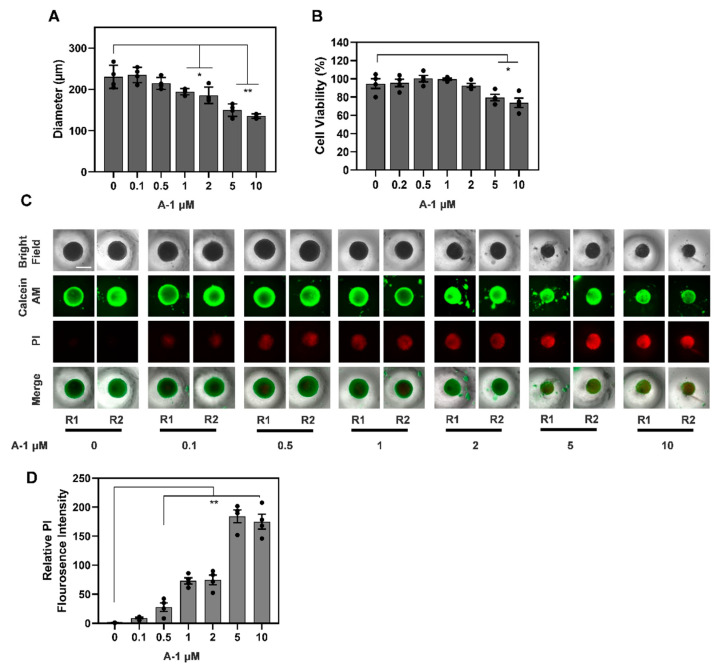
Arachidin−1 (A−1) reduces growth and induces cell death of TNBC MDA−MB−231 spheroids. TNBC MDA-MB-231 spheroids were treated with 0.1, 0.5, 1, 2, 5 and 10 μM A-1 or diluent DMSO (denoted as 0) for 3 days. (**A**) Growth of spheroids treated with different concentrations of A−1 or DMSO was evaluated and represented as Δ diameter between day 3 and day 0. (**B**) Percentage of cell viability of spheroids treated for 3 days with different concentrations of A−1 or DMSO. (**C**) Cell death and viability was evaluated by staining spheroids with calcein AM (green) and PI (red). Two representative spheroids, R1 and R2, are shown for each treatment. Scale bar: 500 μm. (**D**) Cell death was evaluated in spheroids stained with PI and quantified as relative fluorescence intensity of PI in samples treated as shown in (**C**). Data represent mean ± SEM, N = 4; * *p* < 0.05, ** *p* < 0.001, compared to DMSO.

To evaluate the effects of A-1 in human TNBC 3D models, we generated TNBC spheroids by seeding MDA-MB-231 cells for 6 days. The spheroids were treated with varying concentrations of A-1 or DMSO for an additional 3 days. We found that A-1 decreased the TNBC spheroid growth in a dose-dependent manner, with significant reductions of up to 15% and 35% observed at 1 and 5 μM, respectively (Figure 11A). Additionally, the viability of the spheroids significantly decreased at A-1 concentrations of 5 μM and above (Figure 11B). Next, we evaluated if A-1 induced cell death. We found that A-1 induced cell death in TNBC spheroids at concentrations as low as 0.1 μM, with significant cell death observed at concentrations 1 μM and above, as indicated by an increased relative mean fluorescence intensity of PI staining (Figure 11C,D). 

**Figure 12:**
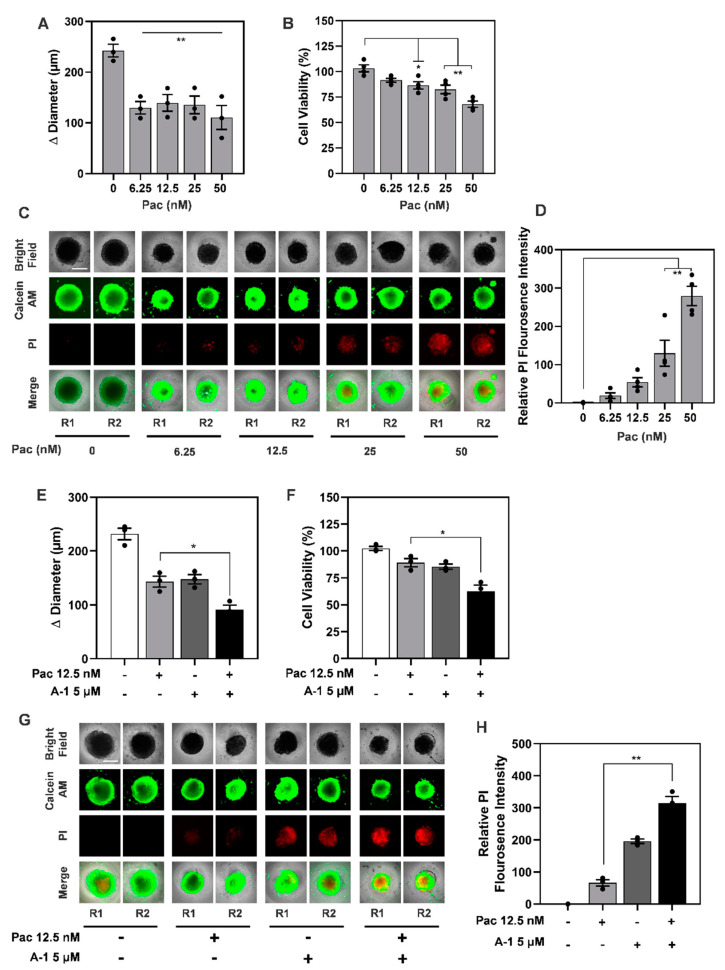
Paclitaxel (Pac) decreases growth of TNBC MDA−MB−231 spheroids and arachidin−1 (A−1) increases the efficacy of Pac in reducing viability in TNBC MDA−MB−231 spheroids. Spheroids were treated with 6.25, 12.5, 25 or 50 nM Pac or diluent DMSO (denoted as 0) for 3 days. (**A**) Growth of spheroids treated with different concentrations of Pac or DMSO was evaluated and represented as Δ diameter between day 3 and day 0. (**B**) Percentage of cell viability of spheroids treated with different concentrations of Pac or DMSO. (**C**) Cell death and viability were evaluated by staining spheroids with calcein AM (green) and PI (red). Two representative spheroids, R1 and R2, shown for each treatment. Scale bar: 500 μm. (**D**) Cell death was evaluated in spheroids stained with PI and quantified as relative fluorescence intensity of PI in samples treated as shown in (**C**). Spheroids were treated with 12.5 nM Pac or 5 μM A−1 alone, in combination or in the presence of diluent DMSO (denoted as −) for 3 days. (**E**) Growth of spheroids treated as described was evaluated and represented as Δ diameter between day 3 and day 0. (**F**) Percentage of cell viability of spheroids treated as described above. (**G**) Cell death and viability were evaluated by staining spheroids with calcein AM (green) and PI (red). Two representative spheroids, R1 and R2, are shown for each treatment. Scale bar: 500 μm. (**H**) Cell death was evaluated in spheroids stained with PI and quantified as relative fluorescence intensity of PI in samples treated as shown in (**E**). All data represent mean ± SEM, N = 3. * *p* < 0.05, ** *p* < 0.001, compared to treatment with Pac alone.

Next, we evaluated the impact of A-1 in combination with Pac treatment on TNBC spheroids. To this end, we first determined the lowest concentration of Pac affecting the spheroid growth and viability. TNBC spheroids were treated with increasing concentrations of Pac ranging from 6.25 to 50 nM for 3 days. We found that Pac significantly diminished spheroid growth at all concentrations tested (Figure 12A). Concomitantly, Pac decreased spheroid viability in a dose-dependent manner with a significant 15% decrease at 12.5 nM (Figure 12B). We also found that Pac induced cell death in TNBC spheroids as indicated by higher relative mean fluorescence intensity of PI staining (Figure 12C,D). Therefore, 12.5 nM Pac was selected as the dose for further experiments since it was the lowest concentration tested that significantly reduced the growth and viability of TNBC spheroids.

To investigate whether A-1 sensitizes TNBC spheroids to Pac, six-day-old spheroids were treated with Pac (12.5 nM), A-1 (5 μM), or with a combination of Pac and A-1 for three days; spheroids treated with DMSO were used as the control. We found that spheroid growth was significantly reduced in spheroids treated with Pac and A-1 compared to either of the single treatments (~45%, Figure 12E). To evaluate the mechanism underlying the decline in spheroid growth observed in the Pac and A-1 combination treatment, we evaluated the effects on cell viability and cell death. The Pac and A-1 combination treatment decreased spheroid viability by ~30% compared to spheroids treated with Pac alone (Figure 12F). Consistent with the significant decrease in cell viability, the combination treatment increased cell death by ~5-fold compared to Pac alone and ~2-fold compared to A-1 alone (Figure 12G,H). Taken together, these findings demonstrate that A-1 increases Pac efficacy by inducing cell death in human TNBC spheroids.

## 4. Discussion

Pac is one of the most effective treatments against TNBC [11]; however, it is not selectively cytotoxic for cancer cells. Additionally, when TNBC cells are repeatedly exposed to Pac, they often develop drug resistance. Therefore, higher dosages of Pac are needed to attain a tumoricidal effect which, in turn, leads to more frequent and severe side effects [24]. These facts underline the necessity for adjuvant therapy options. Natural plant-derived compounds are attracting more attention than synthesized compounds due to their increased safety and efficacy [25]. Several studies have been conducted on the plant-derived compound RES as an adjuvant for chemotherapy drugs due to its low toxicity to normal cells while also effectively sensitizing TNBC cells to chemotherapeutics. However, several studies indicate that RES has low bioavailability. Arachidins are a type of prenylated stilbenoids produced in peanuts. Due to their extra prenyl side chain, arachidins may have increased bioavailability compared to their non-prenylated analogs, such as the most studied stilbene, RES. The potential slowed metabolism of prenylated stilbenoids within the body may lead to improved bioavailability [13].

The main complication when treating TNBC tumor cells is drug resistance. One way to overcome this issue is to find a potent adjuvant to improve the efficacy of chemotherapy drugs such as Pac. In a previous study, A-1 at low concentration (1 µM) arrested the cell cycle at G2/M phase and induced apoptosis through the intrinsic pathway in both MDA-MB-231 and MDA-MB-436 TNBC cells. In this previous study and herein, we observed that RES, A-3, and A-1 inhibited cell proliferation; however, the IC_50_ of A-1 was lower than A-3 and RES. This demonstrated that the prenylated stilbenoid A-1 was more cytotoxic when compared to the non-prenylated stilbenoid RES and prenylated stilbenoid A-3. Several studies suggested that RES combined with Pac can enhance the sensitivity of Pac-resistant cancer cell lines [26,27]. In testing combination treatments of Pac with the prenylated stilbenoids A-1 and A-3, it was observed that the IC_50_ of Pac decreased in these combination treatments compared to Pac alone (Table 2 and Table S1). Although the same pattern of decreasing Pac IC_50_ was observed in all three combination treatments, higher concentrations of RES and A-3 were needed to produce this pattern. In addition, Pac did not show a significant synergistic effect with RES and A-3 (Table 1 and Table S1). Combination treatments of Pac with A-1 or RES resulted in ~2-fold or greater decrease in the IC_50_ of Pac compared to Pac treatment alone, but only the A-1 and RES combination showed a significant decrease in the IC_50_ (Table 1, Table 2 and Table S1). Similarly, it was previously reported that RES (30 μM and 50 μM) in combination with Pac (2 nM) resulted in a significant reduction in the IC_50_ value of Pac in MDA-MB-231 [26]. Another study showed that high concentrations of RES decreased the IC_50_ of Pac without any change in IC_50_ of RES; however, the lower concentrations of RES decreased the IC_50_ of both Pac and RES [27]. Furthermore, another study showed that Pac (50 μM) combined with RES (50 μM) had a 0.8-fold and 0.7-fold decrease in cell proliferation when compared to Pac and RES alone, respectively [28]. In contrast, some studies show better results using lower concentrations of these compounds [27]. In our investigation of the apoptosis induction by combination treatment, we found significant caspase-3/7 activity in both TNBC cells after treatment with A-1 combined with Pac. Our results agree with previous observations that RES combined with Pac increases apoptosis activity in MDA-MB-231 and DBTRG glioblastoma cells [27,28]. The results from the present study indicate that the IC_50_ values of A-1 and Pac in both TNBC cells were approximately 2 μM and 2 nM, respectively. Additionally, the combination of A-1 (2 μM) and Pac (2 nM) as well as the 10-fold lower concentrations had no significant cytotoxic effect on non-cancerous cell line MCF-10A (Figure 3). In agreement with our data, a previous paper showed that A-1, at low concentrations (2 μM and 5 μM), had prooxidant effects on cancer cells (MDA-MB-231, MDA-MB-436, and human leukemia HL-60 cells); however, A-1 had no cytotoxic effect on MCF-10A [14,19]. Therefore, we focused on the low concentrations of A-1 (0.2 μM and 2 μM) combined with low concentrations of Pac (0.2 nM and 2 nM). 

A key marker for cellular apoptosis induction is ROS accumulation; this stimulates the ROS-dependent apoptosis signaling pathways. Our results showed that intracellular ROS levels increased after treatment with A-1 (0.2 µM) from 6 h to 48 h, when compared to controls, but each TNBC cell had a different increase pattern. In MDA-MB-231, the ROS level increased immediately after treatment; however, in MDA-MB-436, the ROS level increased steadily over the 12 h to 48 h period (Figure 4). Additionally, the results showed that the combination treatment of cells with A-1 and Pac enhanced the ROS activity more in MDA-MB-231 (by ~2-fold) than MDA-MB-436 (by less than ~1-fold) in comparison with both single treatments (Figure 5A,B). Previous studies have shown that both epithelial TNBC cells, MDA-MB-231 and MDA-MB-436, with mesenchymal-like phenotypes are mechanically different from each other, with MDA-MB-231 being more aggressive than MDA-MB-436 [29]. Their cluster data analysis suggested that MDA-MB-231 cell lines are indeed more different from MDA-MB-436 cell lines than from the non-tumorigenic MCF-10A cell. These findings suggest the reason for the unique ROS accumulation timelines observed in these two TNBC cell lines [29]. Due to the connection between ROS accumulation and caspase induction, the previous discussion demonstrated that MDA-MB-231 and MDA-MB-436 activate caspases in different time patterns [30]. 

Increased ROS activity also activates tumor suppressor protein p53, which controls the cellular stress responses. These stress responses can induce cell cycle arrest in order to repair DNA or cause cell death by apoptosis. Generally, p53 controls the expression of potent cyclin-dependent kinase (CDK) inhibitors that prevent the uncontrolled cell cycle progression which leads to cancerous cell growth [31]. In this study, A-1 combined with Pac created a large population of cells in cell cycle arrest at the G2/M phases after 24 h of treatment when compared to 24 h treatments for either A-1 or Pac alone (Figure 6). In other words, A-1 in combination with Pac effectively inhibits the proliferation of cancer cells by accumulating cells in G2/M phase. It has been observed that RES arrests cells in G1 phase in MDA-MB-231 cells while A-1 alone and Pac alone arrest cells in G2/M phase after 24 h treatment for the same cell line [32]. This finding was also observed in other studies; for example, one study showed that the combination of docetaxel (1 nM) with RES (15 µM) arrests cells in G2/M phase, while RES alone did not induce a significant G2/M arrest in breast cancer cell lines (SK-BR-3, MCF-7, MDA-MB-231, and T47D) [33]. Another study suggested that polydatin (1 µM, 2.5 µM and 5 µM), a glycoside of RES, arrests cells in S-phase in cancer cell lines MDA-MB-231 and MCF-7 [34]. In addition, low concentrations of Z-DAN-11 ((Z)-2, 3-diaryl acrylonitrile (trans-stilbene) derivatives; 2.5 µM, 5 µM, and 10 µM), a similar trans-stilbene compound, arrested cells in G2/M phase in cancer cell lines MCF-7 and A549 [35]. Furthermore, RES (25 µM) combined with Pac (2 nM) inhibits cell populations in G2/M phase cell cycle in 5637 bladder human cancer cells [36]. Docetaxel achieves its therapeutic efficacy by inhibiting the depolymerization of tubulin and thereby inducing cell cycle arrest. 

Based on our results, A-1 combined with Pac inhibits the proliferation of cancer cells by accumulating cells in G2/M phase. To support our results, we monitored p53 expression levels. Both cell lines showed increased expression of p53 upon treatment with A-1 in combination with Pac (Figure 7). Our results agree with previous observations that p53 was upregulated in Pac (10 nM and 20 nM) or RES (12.5 µM, 25 µM, and 50 µM)-treated TNBC cells [37,38,39]. Therefore, we hypothesize that p53 activation was caused by ROS induction.

Additionally, we hypothesized that activation of caspase-3 and caspase-7, directly and indirectly, will result in the breakdown of cellular proteins such as the nuclear matrix and cytoskeleton. Thus, at this stage, phosphatidylserine (PS)—a protein marker of apoptosis—will be displayed on the outer layer of the cellular membrane and will be detected by Annexin V staining. The present study suggested that both cell lines, after 24 h treatment with A-1 in combination with Pac, will have increased percentages of cell populations detected with Annexin V, indicating early apoptosis (Figure 8). The results were supported by a study in which Pac (10 nM) induced apoptosis by shifting the cell population into early apoptosis in cancer cell lines MDA-MB-231 and MCF-7 [37]. 

**Figure 13:**
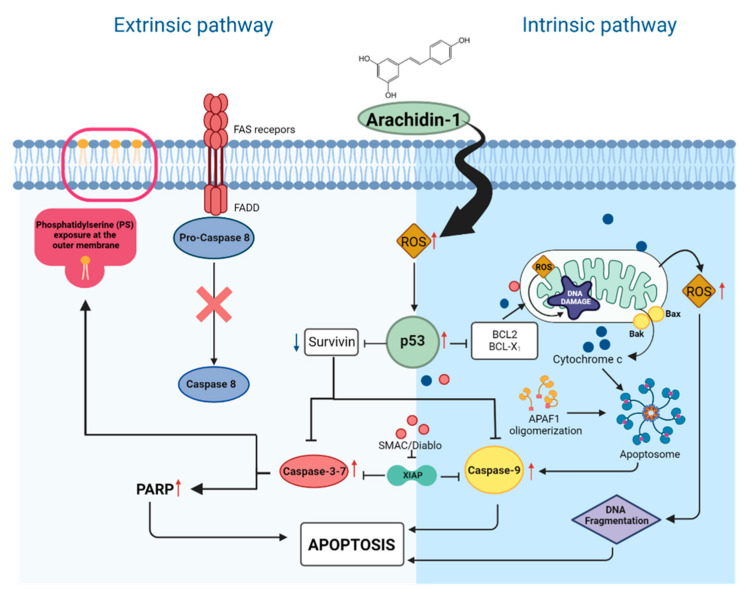
Schematic representation of targeting various intracellular signaling pathways using arachidin-1 as a novel therapeutic strategy in the treatment of triple-negative breast cancer. Red and blue arrows represent up- and down-regulation, respectively.

With consideration of our above findings that A-1 combined with Pac induces apoptosis, we wanted to better understand the molecular intricacies leading to the apoptotic outcome. To observe this, we measured the expression of various apoptosis-related caspases. The caspase cascade is key for the induction of both the extrinsic and intrinsic apoptosis pathways. Specifically, caspase-8 is associated with the activation of the extrinsic pathway, while caspase-9 is associated with intrinsic (mitochondrial) pathway activation [40]. Several studies presented that Pac alone and RES alone have been reported to induce apoptosis through the activation of caspase-9 in breast cancer cells [32,41]. Pac alone or in combination with RES activates caspase-7 in MDA-MB-231 [27,42]. Thus, our findings agree with previous studies demonstrating that the expression levels of caspase-8 were not notably altered after treatment with Pac alone or in combination with A-1 when compared to the control at either time point in both cell lines. However, the expression levels of cleaved caspase-9 and full-length caspase-7 were increased in MDA-MB-231 after treatment with A-1 combined with Pac in comparison to single treatments and controls (Figure 9C,D and Figure 11). In MDA-MB-436, combination treatment significantly reduced the expression level of full-length caspase-9 and significantly increased the expression levels of caspase-7 in comparison to single treatment and control group (Figure 10C,D). Based on our results, the mechanism of action for A-1 combined with Pac-induced apoptosis was dependent on survivin expression. In contrast, the previous study showed that RES in high concentrations (300 µM) correlated with a reduction in survivin expression in MDA-MB-231 Pac-sensitive and MDA-MB-231 Pac-resistant cells [27]. These results may indicate that the combination of A-1 with Pac potentially induces apoptosis in TNBC cell lines through the intrinsic pathway (Figure 13). Most studies, however, have relied on 2D culture models, which do not fully translate to clinical settings since they are unable to accurately simulate the complicated nature of tumor architecture. However, significant findings show that three-dimensional (3D) tumor spheroids were superior to other preclinical models for assessing medication efficacy. In this study, we established MDA-MB-231 spheroids, investigated the effects of A-1 combined with Pac, and compared this to both single treatments. Herein, our 3D results, in agreement with our 2D results, showed that A-1 increased the effectiveness of Pac by inducing cell death in human TNBC spheroids in comparison to A-1 treatment alone. Thus, our findings open a new door to investigate this combination treatment in more depth.

## 5. Conclusions

In conclusion, this study evaluated the synergic effects of A-1 and Pac treatment within a 2D MDA-MB-231 and MDA-MB-436 cell culture model as well as a 3D TNBC spheroid model. After assessing several parameters within the 2D and 3D models, the results illustrated that A-1 in low micromolar concentrations enhances the chemotherapeutic drug potential of Pac. The various cell apoptosis assays used throughout this study suggested that the A-1 and Pac combination treatment may increase the anticancer effects of these compounds through synergistic interactions. These interactions induce apoptosis through the intrinsic apoptosis pathway, which is dependent on oxidative stress. Moreover, our findings suggest that A-1 combined with Pac has the potential to function as a novel plant-derived treatment for triple-negative breast cancer. 

## Figures and Tables

**Table 1:** Cytotoxicity of resveratrol (RES) and paclitaxel (Pac) alone or in combination in TNBC cells.

**24 h**	**RES (µM)**	**Pac IC_50_ (nM)**	**Fold Decrease Compared to Pac Alone**	**Pac (nM)**	**RES IC_50_ (µM)**	**Fold Decrease Compared to RES Alone**
**MDA-MB-231**	0	2.69 ± 0.13		0	10.67 ± 0.35	
	0.1	1.97 ± 0.25	0.96	0.1	7.94 ± 1.45	1.30
	1	1.11 ± 0.23 *	1.71	1	6.68 ± 1.34	1.55
	10	0.42 ± 0.02 **	4.53	10	6.46 ± 1.24	1.60
**MDA-MB-436**	0	2.47 ± 0.28		0	7.14 ± 2.13	
	0.1	1.59 ± 0.12	1.80	0.1	9.07 ± 0.31	1.10
	1	1.20 ± 0.32 *	2.37	1	8.52 ± 1.14	1.17
	10	0.37 ± 0.04 **	7.78	10	6.16 ± 3.49	1.62
**48 h**	**RES (µM)**	**Pac IC_50_ (nM)**	**Fold decrease compared to Pac Alone**	**Pac (nM)**	**RES IC_50_ (µM)**	**Fold decrease compared to RES Alone**
**MDA-MB-231**	0	2.60 ± 0.14		0	10.36 ± 0.74	
	0.1	1.93 ± 0.32	0.98	0.1	7.39 ± 0.96	1.40
	1	0.86 ± 0.19 **	2.22	1	5.14 ± 2.01 **	2.01
	10	0.56 ± 0.14 **	3.37	10	9.09 ± 2.55	1.14
**MDA-MB-436**	0	2.81 ± 0.44		0	9.53 ± 0.12	
	0.1	1.54 ± 0.11	1.85	0.1	10.04 ± 0.67	0.99
	1	0.90 ± 0.19 **	3.17	1	8.08 ± 2.17	1.24
	10	0.61 ± 0.20 **	4.64	10	4.57 ± 1.11 *	2.19
**72 h**	**RES (µM)**	**Pac IC_50_ (nM)**	**Fold decrease compared to Pac Alone**	**Pac (nM)**	**RES IC_50_ (µM)**	**Fold decrease compared to RES Alone**
**MDA-MB-231**	0	1.90 ± 0.39		0	10.33 ± 0.64	
	0.1	1.15 ± 0.32	1.65	0.1	10.83 ± 0.92	0.95
	1	0.68 ± 0.16	2.79	1	6.65 ± 1.17	1.55
	10	0.33 ± 0.01 *	5.75	10	11.82 ± 5.30	0.87
**MDA-MB-436**	0	2.85 ± 0.17		0	9.99 ± 1.19	
	0.1	1.82 ± 0.24	1.56	0.1	10.16 ± 0.83	0.98
	1	1.40 ± 0.07	2.04	1	9.99 ± 0.54	1.00
	10	0.70 ± 0.13 *	4.06	10	6.70 ± 1.38	1.49

IC_50_ values of paclitaxel, resveratrol, and the IC_50_ fold decrease compared to each compound alone for all combinations ± the standard error of mean (* *p* < 0.05; ** *p* < 0.01; * *p*-values compare IC_50_ values of combination treatment to individual compound alone).

**Table 2:** Cytotoxicity of arachidin−1 (A−1) and paclitaxel (Pac) alone or in combination in TNBC cells.

**24 h**	**A-1** **(µM)**	**Pac IC_50_** **(nM)**	**Fold decrease Compared to Pac Alone**	**Pac (nM)**	**A-1 IC_50_ (µM)**	**Fold Decrease Compared to A-1 Alone**
**MDA-MB-231**	0	2.20 ± 0.12		0	2.71 ± 0.06	
	0.1	1.15 ± 0.11 **	1.95	0.1	1.85 ± 0.38	1.80
	1	0.75 ± 0.21 **	2.98	1	0.70 ± 0.27 **	4.72
	10	0.39 ± 0.14 **	5.72	10	0.66 ± 0.03 ****	5.04
**MDA-MB-436**	0	2.24 ± 0.11		0	2.72 ± 0.14	
	0.1	1.04 ± 0.01 ***	2.14	0.1	1.60 ± 0.25 *	1.34
	1	0.60 ± 0.03 **	3.68	1	0.86 ± 0.34 **	2.50
	10	1.78 ± 0.37 *	1.25	10	0.45 ± 0.01 ****	4.78
**48 h**	**A-1** **(µM)**	**Pac IC_50_** **(nM)**	**Fold decrease compared to Pac Alone**	**Pac (nM)**	**A-1 IC_50_ (µM)**	**Fold decrease compared to A-1 Alone**
**MDA-MB-231**	0	2.21 ± 0.23		0	2.71 ± 0.17	
	0.1	1.44 ± 0.09	1.56	0.1	1.94 ± 0.36	1.72
	1	1.20 ± 0.10 *	1.87	1	0.88 ± 0.08 **	3.76
	10	1.10 ± 0.12 *	2.04	10	0.58 ± 0.19 **	5.72
**MDA-MB-436**	0	2.27 ± 0.05		0	2.36 ± 0.19	
	0.1	1.30 ± 0.05 ***	1.70	0.1	1.17 ± 0.12 *	1.85
	1	0.85 ± 0.06 ***	2.60	1	0.77 ± 0.07 **	2.78
	10	1.01 ± 0.06 ***	2.20	10	0.88 ± 0.38 *	2.45
**72 h**	**A-1** **(µM)**	**Pac IC_50_** **(nM)**	**Fold decrease compared to Pac Alone**	**Pac (nM)**	**A-1 IC_50_ (µM)**	**Fold decrease compared to A-1 Alone**
**MDA-MB-231**	0	2.25 ± 0.19		0	3.33 ± 0.56	
	0.1	1.30 ± 0.05 *	1.73	0.1	2.34 ± 0.48	1.42
	1	1.08 ± 0.13 *	2.09	1	1.03 ± 0.01 *	3.22
	10	0.70 ± 0.12 **	3.22	10	0.84 ± 0.07 *	3.93
**MDA-MB-436**	0	2.22 ± 0.12		0	2.15 ± 0.09	
	0.1	1.48 ± 0.18	1.50	0.1	1.71 ± 0.22	1.26
	1	0.10 ± 0.04 **	2.22	1	0.87 ± 0.12 **	2.47
	10	0.81 ± 0.20 **	2.73	10	0.72 ± 0.14 **	2.99

IC_50_ values of paclitaxel, arachidin-1, and the IC_50_ fold decrease compared to each compound alone for all combination treatments ± the standard error of mean (* *p* < 0.05; ** *p* < 0.01; *** *p* < 0.001; **** *p* < 0.0001; * *p*-values compare IC_50_ values of combination treatment to individual compound alone).

## Data Availability

The data from this study are available upon request.

## Supplementary Materials

The following supporting information can be downloaded at: https://www.mdpi.com/article/10.3390/cancers15020399/s1, Table S1: Cytotoxicity of arachidin-3 and paclitaxel alone or in combination in TNBC cells. Figure S1: Cytotoxicity of arachidin-1 (A-1, 1 mM) combined with paclitaxel (Pac, 1 nM) in MDA-MB-231 and MDA-MB-436 cell line.; Figure S2: Levels of DCFDA fluorescence normalized to control (DMSO, 0.01%) in TNBC cell line MDA-MB-231 in the absence or presence of H_2_DCFDA; Figure S3: Densitometric protein expression comparison of caspase-7 in TNBC cell lines MDA-MB-231 (A) and MDA-MB-436 (B). File S1: Original blots.
