# Supplementary material for: Arachidin-1, a Prenylated Stilbenoid from Peanut, Enhances the Anticancer Effects of Paclitaxel in Triple-Negative Breast Cancer Cells
Source: Cancers (Basel). 2023 Jan 7;15(2):399. doi: 10.3390/cancers15020399 (PMC9856928; doi:10.3390/cancers15020399)
Supplement: Supplementary file 1 [file cancers-15-00399-s001.zip › cancers-2126416-supplementary.pdf]

Supplementary Information

Table S1. Cytotoxicity of arachidin-3 and paclitaxel alone or in combination in TNBC cells.

**24 h**	**Arachidin-3 (μM)**	**Paclitaxel IC50 (nM)**	**Fold decrease compared to paclitaxel Alone**	**Paclitaxel (nM)**	**Arachidin-3 IC50 (μM)**	**Fold decrease compared to Arachidin-3 Alone**
**MDA-MB-231**	0	2.24 ± 0.03		0	12.78 ± 1.65	
	0.1	1.14 ± 0.12 **	2.04	0.1	9.24 ± 1.73	1.36
	1	0.78 ± 0.06 ****	2.99	1	7.11 ± 0.11 *	1.77
	10	0.60 ± 0.19 **	3.98	10	6.72 ± 0.18 *	1.88
**MDA-MB-436**	0	2.87 ± 0.17		0	12.78 ± 1.65	
	0.1	2.40 ± 0.19	1.03	0.1	9.24 ± 1.73	1.19
	1	1.29 ± 0.07 **	1.92	1	7.11 ± 0.11 *	1.55
	10	0.55 ± 0.19 **	4.54	10	6.72 ± 0.18 *	1.65
**48 h**	**Arachidin-3 (μM)**	**Paclitaxel IC50 (nM)**	**Fold decrease compared to paclitaxel Alone**	**Paclitaxel (nM)**	**Arachidin-3 IC50 (μM)**	**Fold decrease compared to Arachidin-3 Alone**
**MDA-MB-231**	0	2.52 ± 0.07		0	14.70 ± 1.79	
	0.1	1.24 ± 0.10 **	1.89	0.1	9.86 ± 1.92	1.28
	1	0.73 ± 0.14 ***	3.19	1	7.67 ± 1.82	1.64
	10	0.79 ± 0.05 ****	2.95	10	7.03 ± 1.21 *	1.79
**MDA-MB-436**	0	2.39 ± 0.27		0	12.98 ± 1.19	
	0.1	2.14 ± 0.11	1.16	0.1	12.45 ± 1.65	0.88
	1	1.45 ± 0.18	1.71	1	6.72 ± 1.03 *	1.64
	10	0.64 ± 0.13	3.85	10	6.36 ± 1.02 *	1.73
**72 h**	**Arachidin-3 (μM)**	**Paclitaxel IC50 (nM)**	**Fold decrease compared to paclitaxel Alone**	**Paclitaxel (nM)**	**Arachidin-3 IC50 (μM)**	**Fold decrease compared to Arachidin-3 Alone**
**MDA-MB-231**	0	2.33 ± 0.18		0	12.60 ± 2.10	
	0.1	0.87 ± 0.19 *	2.68	0.1	10.33 ± 1.90	1.22
	1	0.70 ± 0.23 *	3.33	1	10.04 ± 1.29	1.25
	10	0.62 ± 0.07 **	3.75	10	3.97 ± 1.53	3.17
**MDA-MB-436**	0	2.48 ± 0.12		0	11.00 ± 0.25	
	0.1	1.53 ± 0.16 *	1.63	0.1	8.46 ± 0.68 *	1.30
	1	1.06 ± 0.05 ***	2.34	1	10.63 ± 1.50	1.03
	10	1.22 ± 0.41	2.03	10	5.92 ± 1.13 *	1.86

IC_{50} values of paclitaxel, arachidin-3 , and the IC_{50} fold decrease compared to each compound alone for all combination ± the standard error of mean (**p*<0.05; ***p*<0.01; **p*-values compare IC_{50} values of combination treatment to individual compound alone).

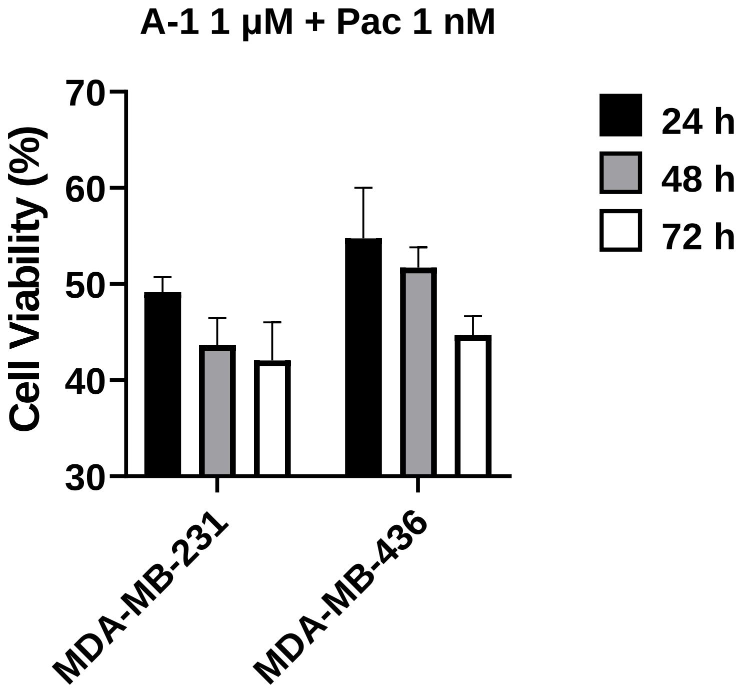

Figure S1. Cytotoxicity of arachidin-1 (A-1, 1 μM) combined with paclitaxel (Pac, 1 nM) in MDA-MB-231 and MDA-MB-436 cell lines. Cell viability was evaluated at 24, 48, and 72 h. Data represent mean ± SEM. N = 3. *p < 0.05 *versus* control.

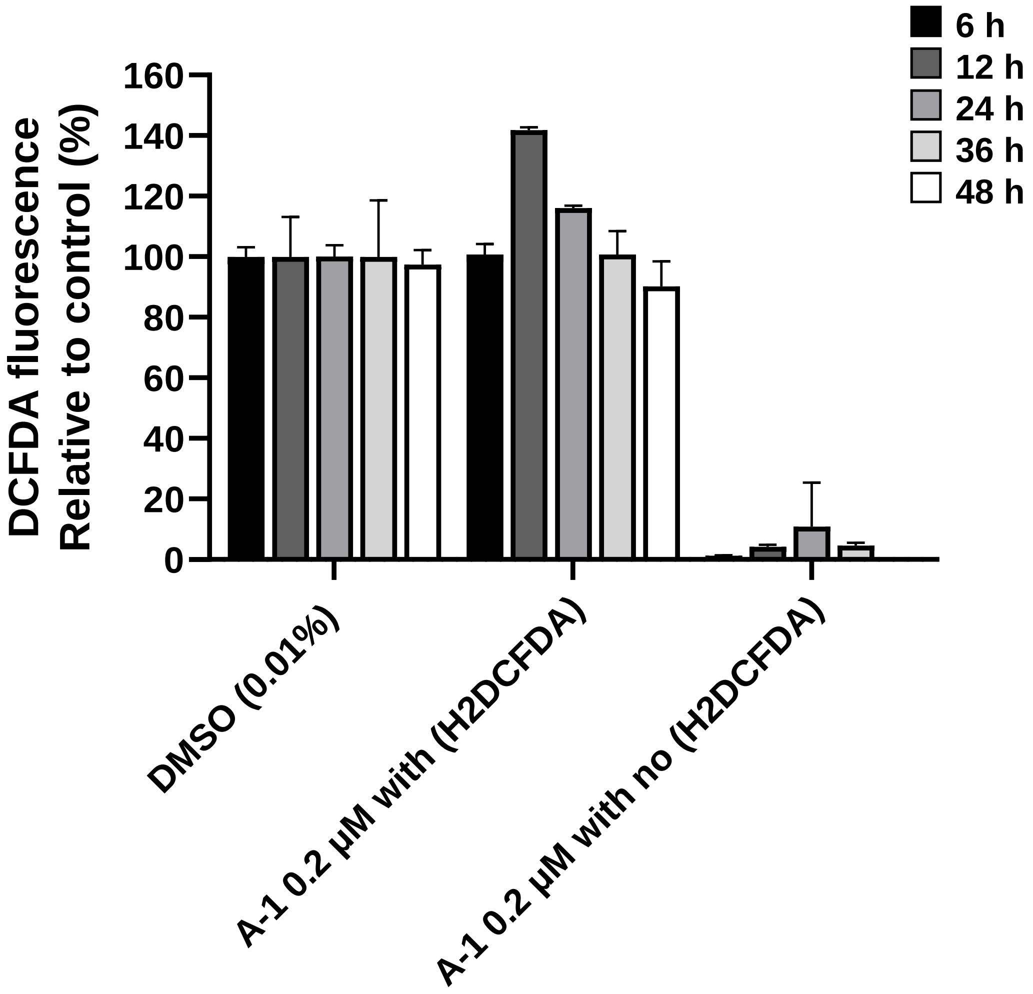

Figure S2. Levels of DCFDA fluorescence normalized to control (DMSO, 0.01%) in TNBC cell line MDA-MB-231 in the absence or presence of H2DCFDA. Fluorescence was measured at excitation: 492–495 nm and emission:517–527 nm. The data is expressed as mean ± SEM of three replicates.

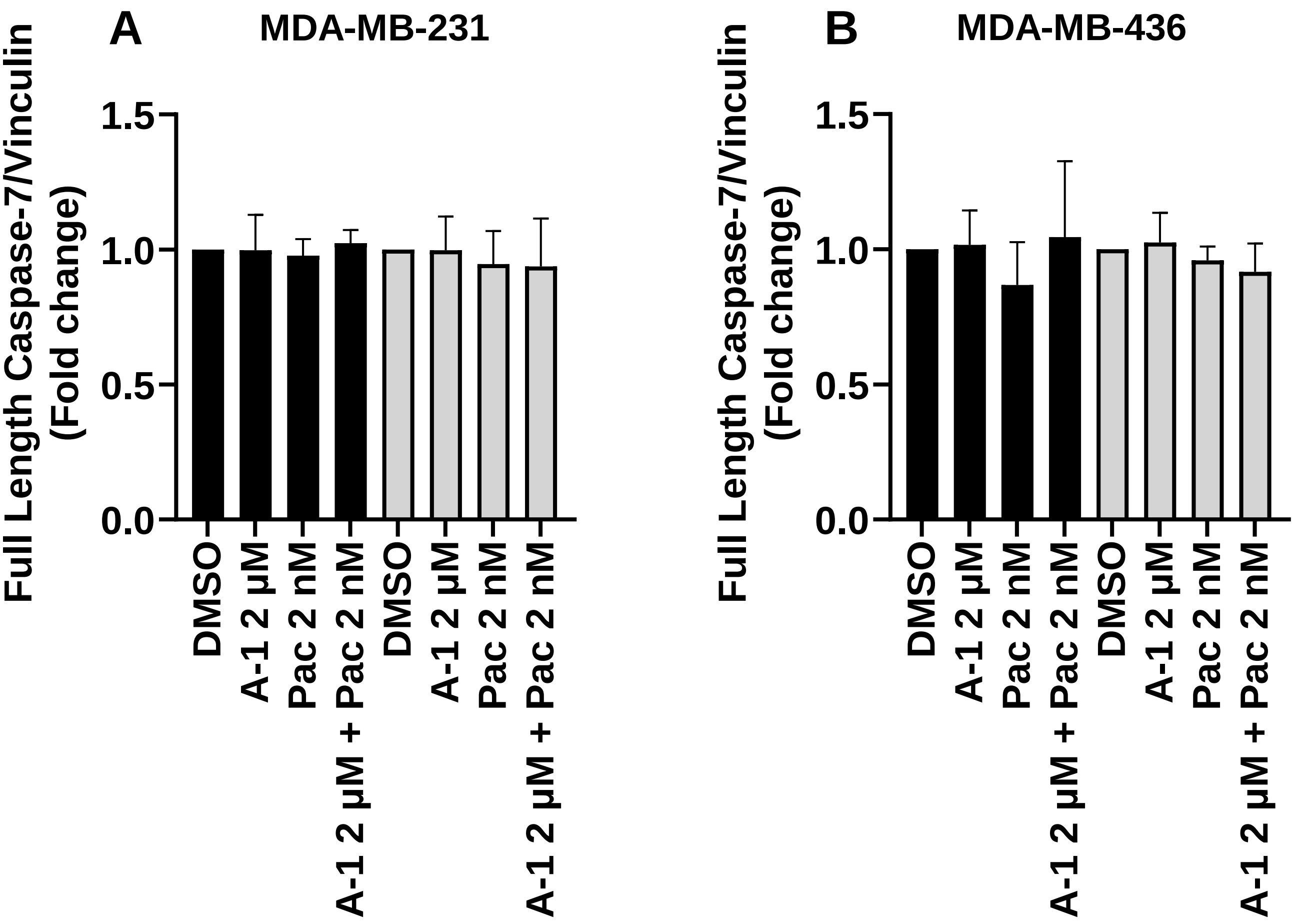

Figure S3. Densitometric protein expression comparison of caspase-7 in TNBC cell lines MDA-MB-231 (A) and MDA-MB-436 (B). Cells were treated with A-1 (0.2 and 2μM) and/or paclitaxel (0.2 and 2 nM) for 24 h (black bars) and 48 h (grey bars). Cells were treated with 0.01% DMSO were used as a control. Densitometric comparisons represent the fold-change of the target protein compared to the loading control vinculin. The data represents mean ± SEM, N = 3.

Western Blot Data

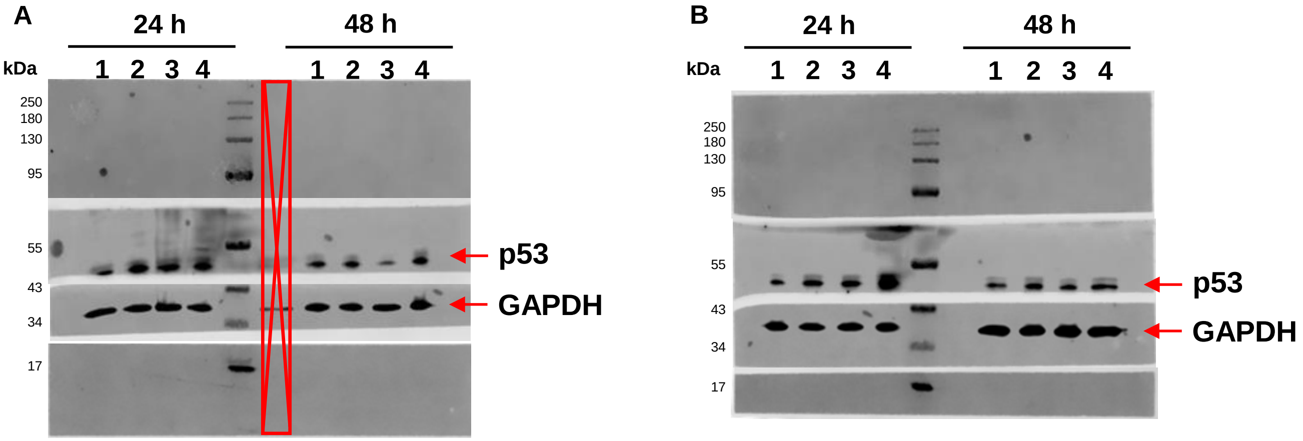

24 h	1	2	3	4
p53/GAPDH	0.44	1.09	1.25	1.81
48 h	1	2	3	4
p53/GAPDH	1.14	1.17	0.47	1.17

24 h	1	2	3	4
p53/GAPDH	0.23	0.54	0.55	1.42
48 h	1	2	3	4
p53/GAPDH	0.81	1.15	0.88	1.14

Original unedited blot used in Figure 7 (A) MDA-MB-231 and (B) MDA-MB-436. 1, DMSO; 2, A-1 2 μM; 3, Pac 2 nM; 4, A-1 2 μM + Pac 2 nM.

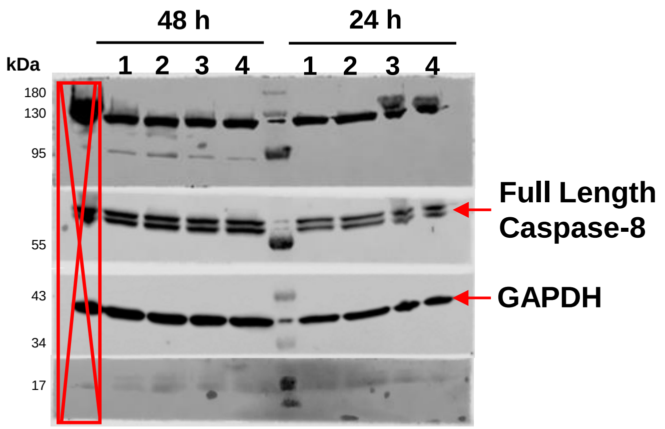

24 h	1	2	3	4
Full Length Caspase-8 /GAPDH	1.64	1.56	1.62	1.43
48 h	1	2	3	4
Full Length Caspase-8 /GAPDH	1.09	1.35	1.29	1.38

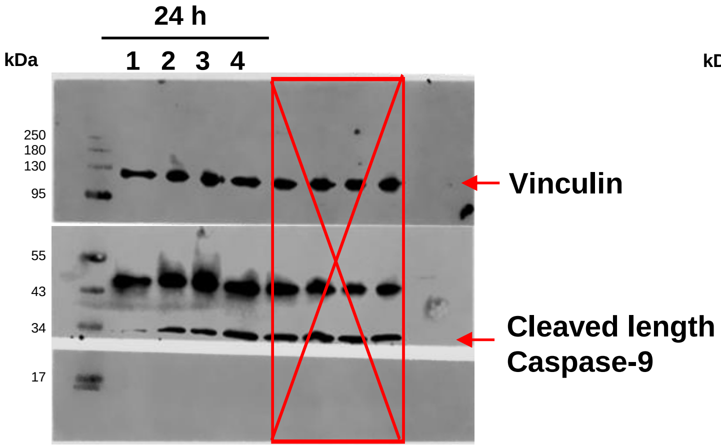

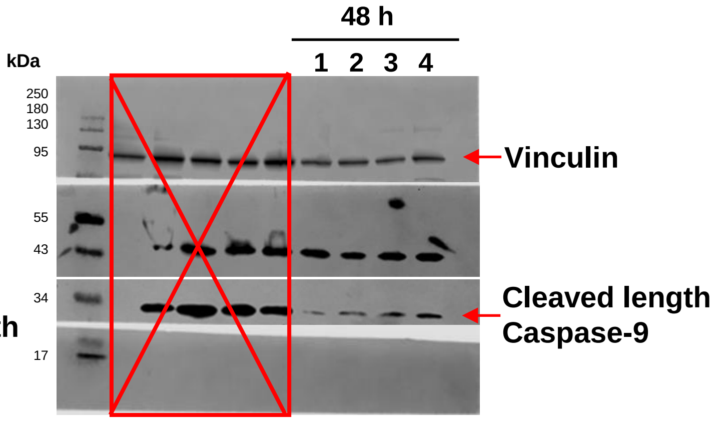

24 h	1	2	3	4
Cleaved Length Caspase-9/Vinculin	0.28	0.78	0.69	1.21

48 h	1	2	3	4
Cleaved Length Caspase-9/Vinculin	0.25	0.65	1.06	0.81

Original unedited blot used in Figure 9. 1, DMSO; 2, A-1 2 μM; 3, Pac 2 nM; 4, A-1 2 μM + Pac 2 nM.

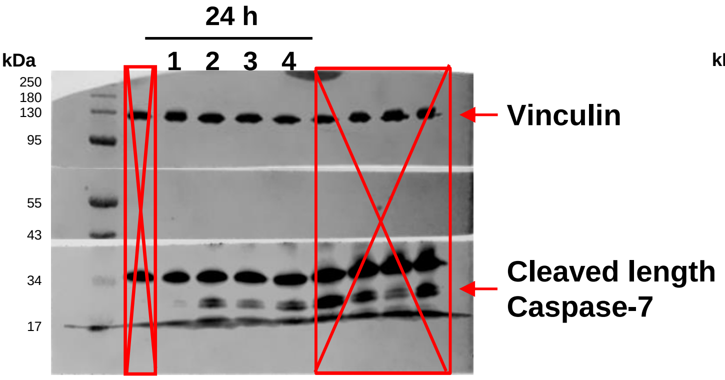

24 h	1	2	3	4
Cleaved length Caspase-7/Vinculin	0.44	1.26	1.02	1.72

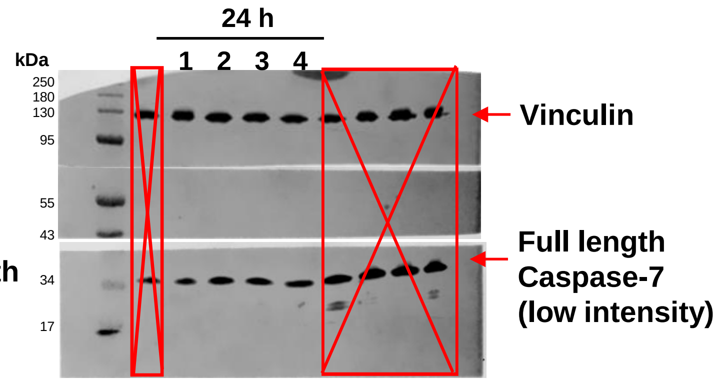

48 h	1	2	3	4
Full length Caspase-7/Vinculin	0.88	1.11	1.15	1.16

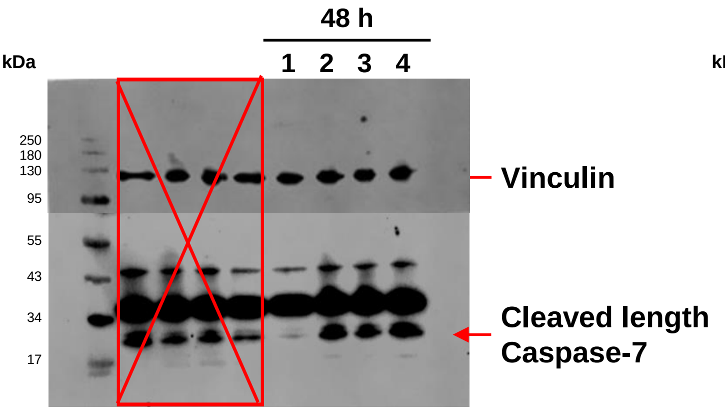

24 h	1	2	3	4
Cleaved length Caspase-7/Vinculin	0.21	1.01	0.86	1.17

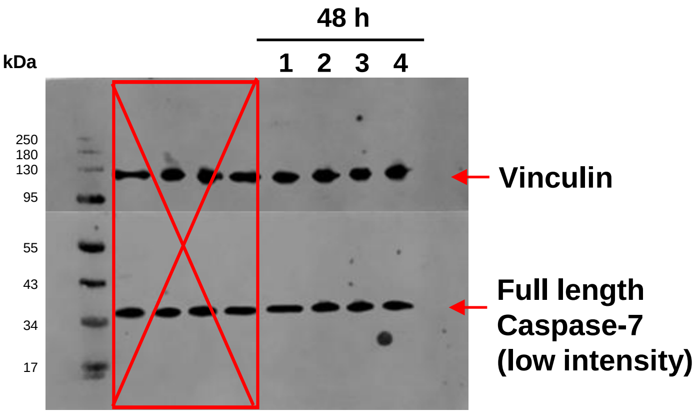

48 h	1	2	3	4
Full length Caspase-7/Vinculin	0.96	0.81	1.00	0.81

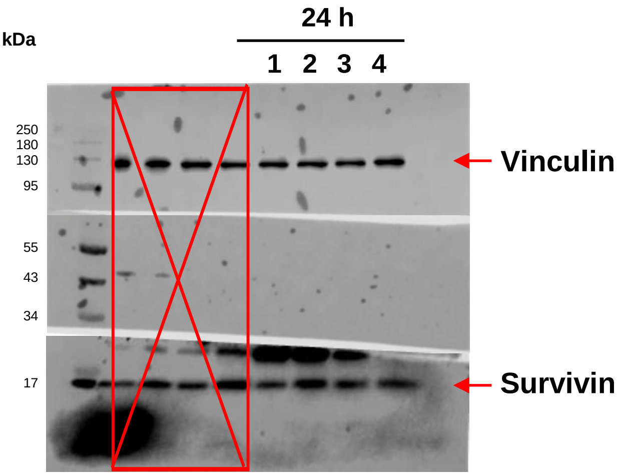

24 h	1	2	3	4
Survivin/Vinculin	1.60	1.19	1.28	0.66

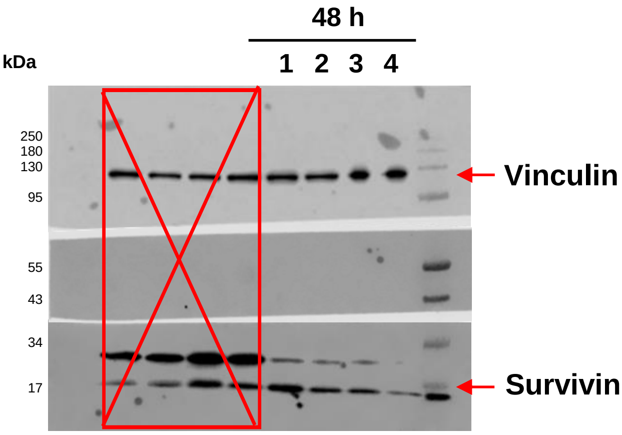

48 h	1	2	3	4
Survivin/Vinculin	1.82	0.98	0.76	0.29

Original unedited blot used in Figure 9. 1, DMSO; 2, A-1 2 μM; 3, Pac 2 nM; 4, A-1 2 μM + Pac 2 nM.

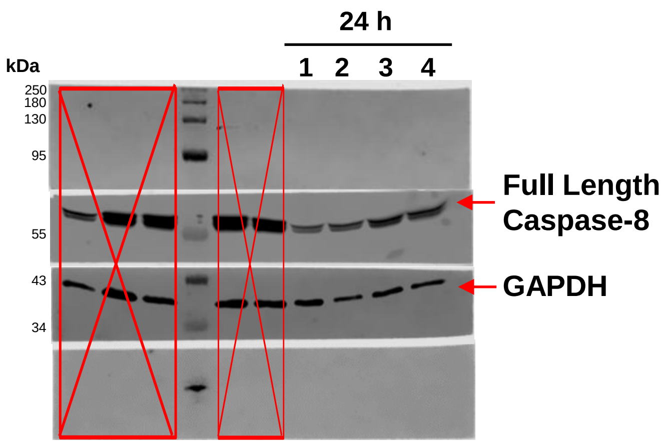

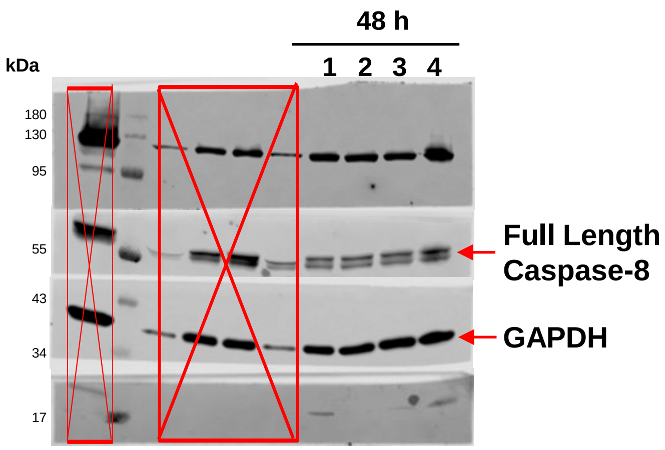

24 h	1	2	3	4
Full Length Caspase-8/GAPDH	0.61	0.64	0.75	0.75

48 h	1	2	3	4
Full Length Caspase-8/GAPDH	1.32	1.24	1.16	1.24

17

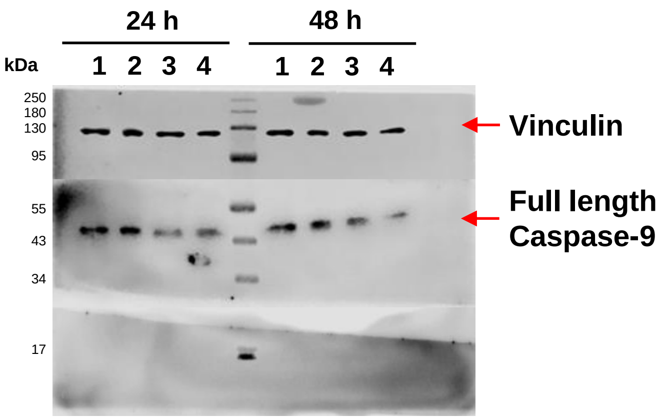

24 h	1	2	3	4
Full Length Caspase-9 /Vinculin	2.75	2.11	0.88	1.20
48 h	1	2	3	4
Full Length Caspase-9 /Vinculin	1.83	1.77	1.34	0.77

Original unedited blot used in Figure 10. 1, DMSO; 2, A-1 2 μM; 3, Pac 2 nM; 4, A-1 2 μM + Pac 2 nM.

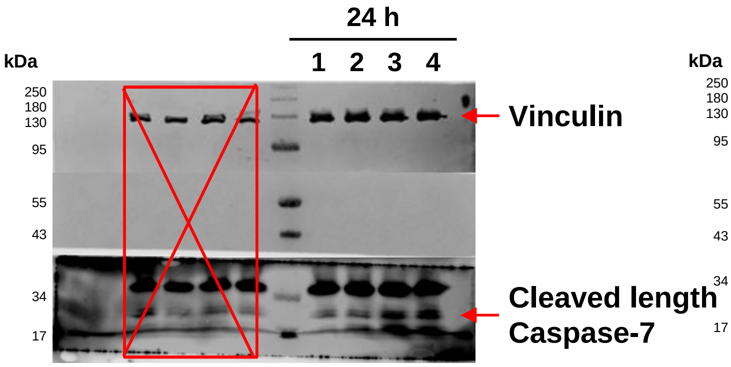

24 h	1	2	3	4
Cleaved length Caspase-7/Vinculin	0.43	0.58	1.45	1.59

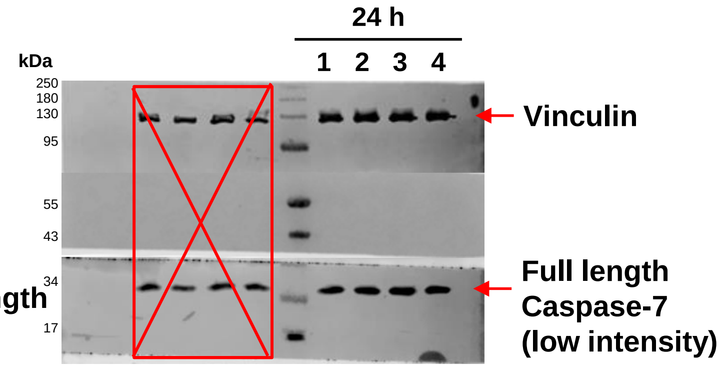

48 h	1	2	3	4
Full length Caspase-7/Vinculin	0.99	0.94	1.11	0.98

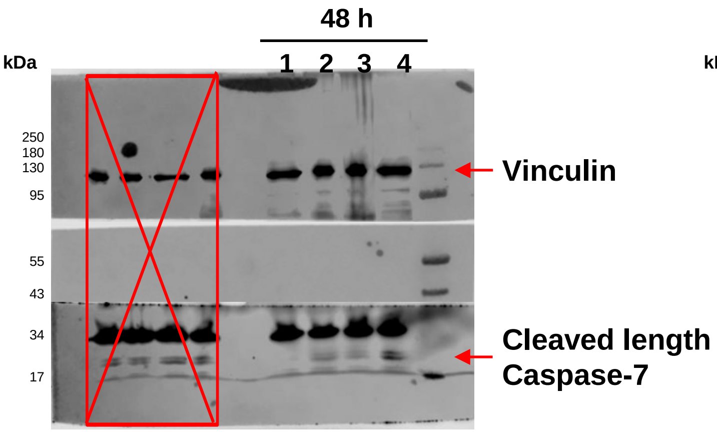

24 h	1	2	3	4
Cleaved length Caspase-7/Vinculin	0.61	0.66	0.54	1.00

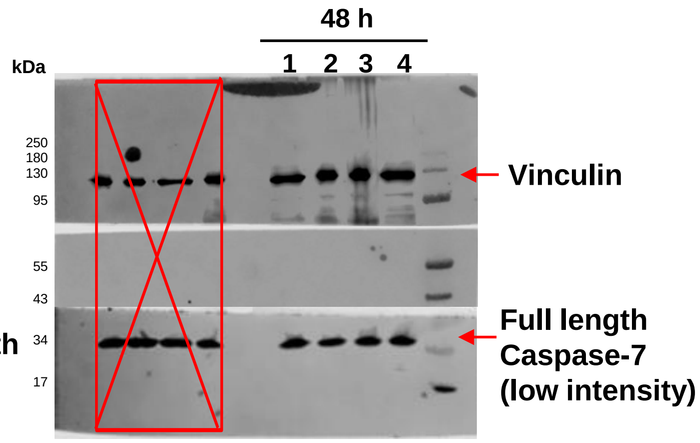

48 h	1	2	3	4
Full length Caspase-7/Vinculin	1.18	1.10	1.13	1.03

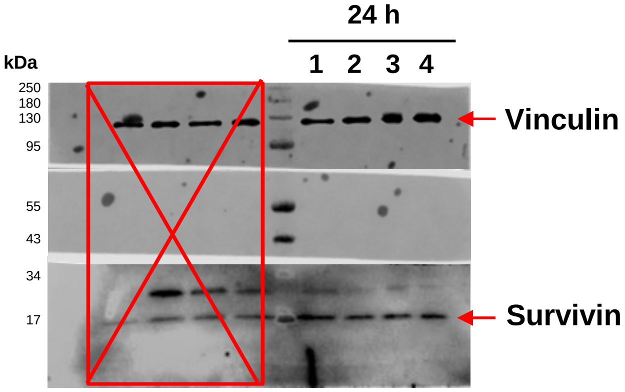

48 h	1	2	3	4
Survivin/Vinculin	2.72	1.20	1.28	0.36

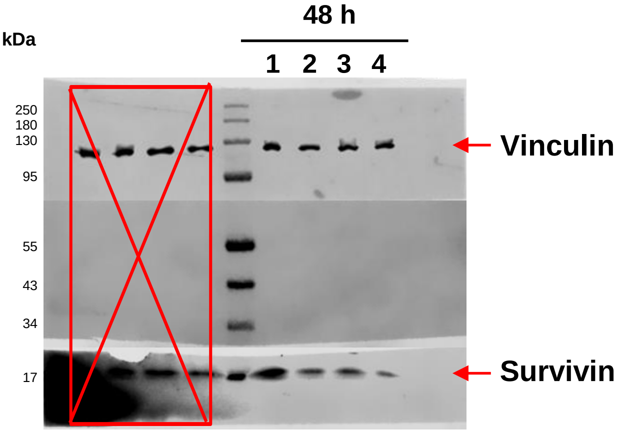

24 h	1	2	3	4
Survivin/Vinculin	1.05	0.33	0.24	0.27

Original unedited blot used in Figure 10. 1, DMSO; 2, A-1 2 μM; 3, Pac 2 nM; 4, A-1 2 μM + Pac 2 nM.
